# Molecular hydrogen in seawater supports growth of diverse marine bacteria

**DOI:** 10.1038/s41564-023-01322-0

**Published:** 2023-02-06

**Authors:** Rachael Lappan, Guy Shelley, Zahra F. Islam, Pok Man Leung, Scott Lockwood, Philipp A. Nauer, Thanavit Jirapanjawat, Gaofeng Ni, Ya-Jou Chen, Adam J. Kessler, Timothy J. Williams, Ricardo Cavicchioli, Federico Baltar, Perran L. M. Cook, Sergio E. Morales, Chris Greening

**Affiliations:** 1 Department of Microbiology, Biomedicine Discovery Institute, Monash University, Melbourne, Victoria Australia; 2 School of Biological Sciences, Monash University, Melbourne, Victoria Australia; 3 Department of Microbiology and Immunology, University of Otago, Dunedin, New Zealand; 4 School of Chemistry, Monash University, Melbourne, Victoria Australia; 5 Centre to Impact AMR, Monash University, Melbourne, Victoria Australia; 6 School of Earth, Atmosphere, and Environment, Monash University, Melbourne, Victoria Australia; 7 School of Biotechnology and Biomolecular Sciences, UNSW Sydney, Sydney, New South Wales Australia; 8 Fungal and Biogeochemical Oceanography, Department of Functional and Evolutionary Ecology, University of Vienna, Vienna, Austria; 9 SAEF: Securing Antarctica’s Environmental Future, Monash University, Melbourne, Victoria Australia

**Keywords:** Microbial ecology, Biogeochemistry

## Abstract

Molecular hydrogen (H_2_) is an abundant and readily accessible energy source in marine systems, but it remains unknown whether marine microbial communities consume this gas. Here we use a suite of approaches to show that marine bacteria consume H_2_ to support growth. Genes for H_2_-uptake hydrogenases are prevalent in global ocean metagenomes, highly expressed in metatranscriptomes and found across eight bacterial phyla. Capacity for H_2_ oxidation increases with depth and decreases with oxygen concentration, suggesting that H_2_ is important in environments with low primary production. Biogeochemical measurements of tropical, temperate and subantarctic waters, and axenic cultures show that marine microbes consume H_2_ supplied at environmentally relevant concentrations, yielding enough cell-specific power to support growth in bacteria with low energy requirements. Conversely, our results indicate that oxidation of carbon monoxide (CO) primarily supports survival. Altogether, H_2_ is a notable energy source for marine bacteria and may influence oceanic ecology and biogeochemistry.

## Abstract (web summary)

Genome-resolved metagenomics, biogeochemistry, modelling and culture-based analysis reveal that marine bacteria consume H_2_ to support growth.

## Main

Over the past decade, trace gases have emerged as major energy sources supporting the growth and survival of aerobic bacteria in terrestrial ecosystems. Two trace gases, molecular hydrogen (H_2_) and carbon monoxide (CO), are particularly dependable substrates given their ubiquity, diffusibility and energy yields^1^. Bacteria oxidize these gases, including below atmospheric concentrations, using group 1 and 2 [NiFe]-hydrogenases and form I carbon monoxide dehydrogenases linked to aerobic respiratory chains^2–6^. Trace gas oxidation enables diverse organoheterotrophic bacteria to survive long-term starvation of their preferred organic growth substrates^7,8^. In addition, various microorganisms can grow mixotrophically by co-oxidizing trace gases with other organic or inorganic energy sources^7,9,10^. Thus far, bacteria from eight different phyla have been experimentally shown to consume H_2_ and CO at ambient levels^1^, with numerous other bacteria encoding the determinants of this process^6,11^. At the ecosystem scale, most bacteria in soil ecosystems harbour genes for trace gas oxidation and cell-specific rates of trace gas oxidation are theoretically sufficient to sustain their survival^12,13^. However, since most of these studies have focused on soil environments or isolates, the wider significance of trace gas oxidation remains largely unexplored.

Trace gases may be important energy sources for oceanic bacteria since they are generally available at elevated concentrations relative to the atmosphere, in contrast to most soils^1^. Surface layers of the world’s oceans are generally supersaturated with H_2_ and CO, typically by 2- to 5-fold (up to 15-fold) and 20- to 200-fold (up to 2,000-fold) relative to the atmosphere, respectively^14–17^. As a result, oceans contribute to net atmospheric emissions of these gases^18,19^. CO is mainly produced through photochemical oxidation of dissolved organic matter^20^, whereas H_2_ is primarily produced by cyanobacterial nitrogen fixation^21^. High concentrations of H_2_ are also produced during fermentation in hypoxic sediments, and these high concentrations can diffuse into the overlying water column, especially in coastal waters^22^. For unresolved reasons, the distributions of these gases vary with latitude and exhibit opposite trends: while dissolved CO is highly supersaturated in polar waters, H_2_ is often undersaturated^23–28^. These variations probably reflect differences in the relative rates of trace gas production and consumption in different climates.

Oceanic microbial communities have long been known to consume CO, although their capacity to use H_2_ has not been systematically evaluated^29^. Approximately a quarter of bacterial cells in oceanic surface waters encode CO dehydrogenases in surface waters and these span a wide range of taxa, including the globally abundant family Rhodobacteraceae (previously known as the marine *Roseobacter* clade)^6,30–33^. Building on observations made for soil communities, CO oxidation potentially enhances the long-term survival of marine bacteria during periods of organic carbon starvation^6^; consistently, culture-based studies indicate that CO does not influence growth of marine isolates, but production of the enzymes responsible is strongly upregulated during starvation^34–37^. While aerobic and anaerobic oxidation of H_2_ has been extensively described in benthic and hydrothermal vent communities^38–42^, so far no studies have shown whether pelagic bacterial communities can use this gas. Several surveys have detected potential H_2_-oxidizing hydrogenases in seawater samples and isolates^6,11,40,43^. Although Cyanobacteria are well-reported to oxidize H_2_, including marine isolates such as *Trichodesmium*, this process is thought to be limited to the endogenous recycling of H_2_ produced by the nitrogenase reaction^44,45^.

In this study, we addressed these knowledge gaps by investigating the processes, distribution, mediators and potential roles of H_2_ and CO oxidation by marine bacteria. To do so, we performed side-by-side metagenomic and biogeochemical profiling of 14 samples collected from a temperate oceanic transect, a temperate coastal transect and a tropical island, in addition to analysing the global *Tara* Oceans metagenomes and metatranscriptomes^46^. We also tested the capacity of three axenic marine bacterial isolates to aerobically consume atmospheric H_2_. Altogether, we provide definitive ecosystem-scale and culture-based evidence that H_2_ is an overlooked key energy source supporting growth of marine bacteria.

## Results

### Marine microbes consume H_2_ slowly and CO rapidly

We measured in situ concentrations and ex situ oxidation rates of H_2_ and CO in 14 surface seawater samples. The samples were collected from three locations (Supplementary Fig. 1): an oceanic transect spanning neritic, subtropical and subantarctic front waters (Munida transect off New Zealand coast; *n* = 8; Supplementary Fig. 2); a temperate urban bay (Port Phillip Bay, Australia; *n* = 4); and a tropical coral cay (Heron Island, Australia; *n* = 2). In line with global trends at these latitudes, both gases were supersaturated relative to the atmosphere in all samples. H_2_ was supersaturated by 5.4-, 4.8- and 12.4-fold respectively in the oceanic transect (2.0 ± 1.2 nM), the temperate bay (1.8 ± 0.26 nM) and the tropical island (4.6 ± 0.3 nM). CO was moderately supersaturated in the oceanic transect (5.2-fold; 0.36 nM ± 0.07 nM), but highly oversaturated in both the temperate bay (123-fold; 8.5 ± 1.7 nM) and tropical island (118-fold; 8.2 ± 0.93 nM).

**Fig. 1:**
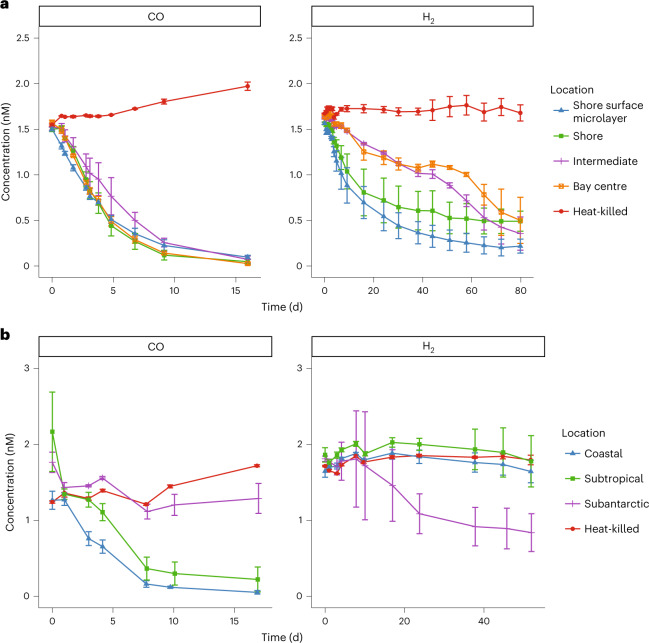
Ex situ oxidation of CO and H_2_ by seawater communities. **a**,**b**, Results are shown for four samples in a transect at Port Phillip Bay, Victoria, Australia (**a**) and eight samples in the Munida transect off the coast of Otago, New Zealand (**b**). Each 120 ml sealed serum vial contained 60 ml of native seawater samples incubated in a 60 ml ambient-air headspace supplemented with ~2.5 ppmv H_2_ or CO. At each timepoint, the mixing ratio of each gas in the headspace of each vial was measured on a gas chromatograph and converted to dissolved gas concentrations (nM). Data are presented as mean ± s.e.m. of three biologically independent samples. Source data

Microbial oxidation of trace gases was detected in all but one of the collected samples during ex situ incubations (Fig. 1). For the temperate bay, H_2_ and CO were consumed in water samples collected from the shore, intermediary zone and bay centre (Fig. 1a). Based on in situ gas concentrations, bulk oxidation rates of CO were 18-fold faster than H_2_ (*P* < 0.0001) (Supplementary Table 1). Bulk oxidation rates did not significantly differ between the surface microlayer (that is, the 1 mm interface between the atmosphere and ocean) and underlying waters. H_2_ and CO oxidation was also evident in surface microlayer and underlying seawater samples collected from the tropical island (Supplementary Fig. 3). We similarly observed rapid CO and slower H_2_ consumption across the multi-front Munida oceanic transect, although unexpectedly, these activities were mutually exclusive. Net CO oxidation occurred throughout the coastal and subtropical waters but was negligible in subantarctic waters. Conversely, net H_2_ oxidation only occurred in the subantarctic waters (Fig. 1b). These divergent oxidation rates in water masses with contrasting physicochemical conditions may help explain the contrasting concentrations of H_2_ and CO in global seawater^23–28^, although wider sampling and in situ assays would be required to confirm this. It should be noted that these measurements probably underestimate rates and overestimate thresholds of H_2_ oxidation since there will still be underlying endogenous production of H_2_, primarily through nitrogen fixation, during the incubations. Nevertheless, they provide the first empirical report of H_2_ oxidation in marine water columns.

### Marine microbes express enzymes for CO and H_2_ oxidation

**Fig. 2:**
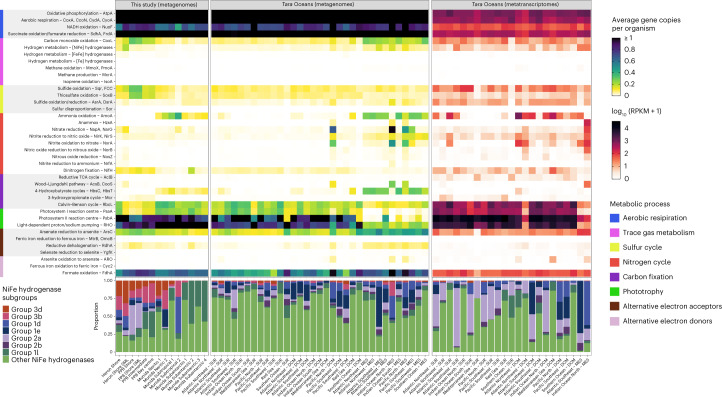
Abundance of metabolic genes encoded by marine communities. The abundance of metabolic marker genes is shown on the basis of the metagenomic short reads across the seawater sampled from the three study sites (left; *n* = 14), metagenomic short reads from the *Tara* Oceans dataset (middle; *n* = 213; replicates averaged) and metatranscriptomic short reads from the *Tara* Oceans dataset (right; *n* = 89; replicates averaged). Homology-based searches were used to calculate the relative abundance of marker genes as average gene copies per organism for the metagenomes (abundance relative to a set of universal single-copy marker genes; equivalent to the estimated proportion of the community encoding a given gene as a single copy) and RPKM for the metatranscriptomes. Where multiple marker genes are listed, values are summed. The bottom panels show the hydrogenase subgroups present in each sample. SUR, surface; DCM, deep chlorophyll maximum; MES, mesopelagic ocean layers. Source data

To better understand the basis of these activities, we sequenced metagenomes of the 14 samples (Supplementary Tables 2 and 3) and used homology-based searches to determine the abundance of 50 metabolic marker genes in the metagenomic reads (Supplementary Table 3) and assemblies (Supplementary Table 4). In common with other surface seawater communities^47^, analysis of community composition (Supplementary Fig. 4) and metabolic genes (Fig. 2) suggests that most bacteria present are capable of aerobic respiration, organoheterotrophy and phototrophy via energy-converting rhodopsins. Capacity for aerobic CO oxidation was moderate: approximately 12% of bacterial and archaeal cells encoded the *coxL* gene (encoding the catalytic subunit of the form I CO dehydrogenase), although relative abundance decreased from an average of 25% in the temperate bay where CO oxidation was highly active to 5.1% in subantarctic waters (Fig. 2) where CO oxidation was negligible (Fig. 1). Diverse hydrogenases were also encoded by the community, including subgroups known to support hydrogenotrophic respiration, hydrogenotrophic carbon fixation, hydrogenogenic fermentation and H_2_ sensing (Supplementary Table 3). Group 1d, 1l and 2a [NiFe]-hydrogenases (herein aerobic H_2_-uptake hydrogenases), which enable cells to input electrons from H_2_ into the aerobic respiratory chain^4,9,48,49^, were by far the most abundant among the H_2_-oxidizing enzymes (Fig. 2). Encoded by 1.0% of marine bacteria on average, the abundance of these hydrogenase subgroups was highest in the tropical island samples (average 3.5%) and declined to 0.11% in the neritic and subtropical samples from the oceanic transect (Fig. 2), in line with the contrasting H_2_ oxidation rates between these samples (Fig. 1 and Supplementary Fig. 3). The dominant hydrogenase subgroups varied between the samples, namely group 1d in the tropical island samples, group 2a in the temperate shore and microlayer samples and group 1l in the subantarctic samples (Fig. 2). Relative abundance of H_2_- and CO-oxidizing bacteria strongly predicted oxidation rates of each gas (*R*^2^ of 0.55 and 0.88; *P* values of 0.0059 and <0.0001, respectively) (Supplementary Fig. 5), although it is likely that repression of gene expression contributes to the negligible activities of some samples.

To test whether these observations were globally representative, we determined the distribution and expression of the genes for H_2_ and CO oxidation in the *Tara* Oceans dataset^47,50^. Similarly to our metagenomes, aerobic H_2_-uptake hydrogenases were encoded by an average of 0.8% bacteria and archaea across the 213 *Tara* Oceans metagenomes, whereas form I CO dehydrogenases were encoded by 10.4%. These genes were observed in samples spanning all four oceans, as well as the Red Sea and Mediterranean Sea (Fig. 2). Despite their relatively low abundance based on the metagenomes, hydrogenase transcripts were highly numerous in the metatranscriptomes, with comparable levels to nitrogenase (*nifH*) transcripts (Fig. 2 and Supplementary Table 3). Expression ratios (average RNA:DNA ratios) of the aerobic H_2_-uptake hydrogenases were high, that is, 2.2, 1.1 and 12.9 for the group 1d, 1l and 2a [NiFe]-hydrogenases, respectively (Supplementary Table 3); of the marker genes surveyed, only the determinants of phototrophy (*psaA*, *psbA*, energy-converting rhodopsins), nitrification (*amoA*, *nxrA*) and CO_2_ fixation (*rbcL*) were expressed at higher ratios than the group 2a [NiFe]-hydrogenases. In contrast, expression levels were relatively low for the CO dehydrogenase (0.9), as well as the hydrogenases responsible for hydrogenotrophic carbon fixation, hydrogenogenic fermentation and H_2_ sensing (average RNA/DNA <1 in all cases) (Supplementary Table 3). Together with the biogeochemical measurements (Fig. 1), these findings suggest that H_2_-oxidizing bacteria can be highly active in seawater despite their relatively low abundance.

### Eleven marine bacterial phyla encode H_2_-oxidizing enzymes

**Fig. 3:**
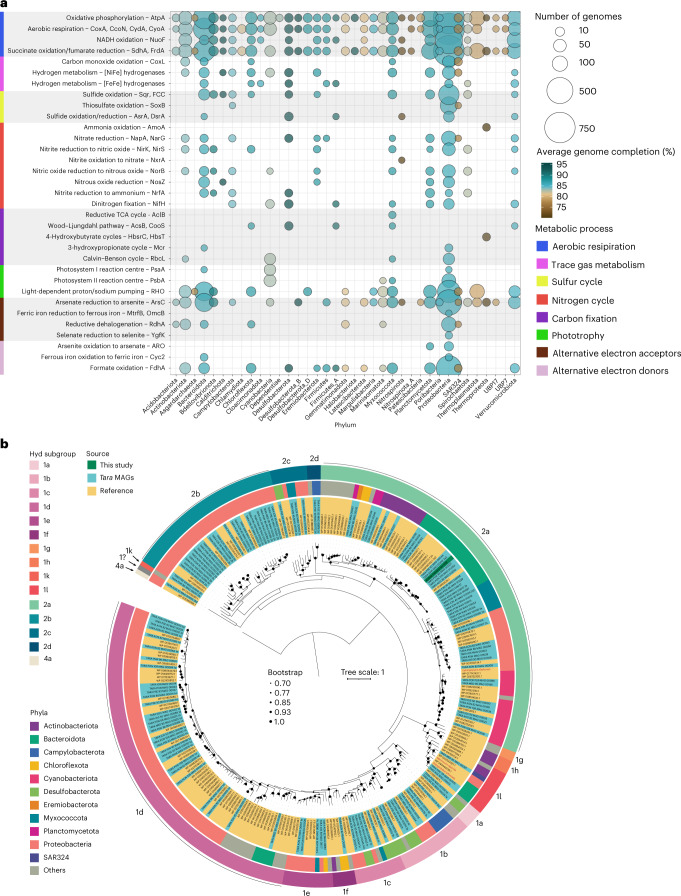
Distribution of metabolic genes in marine bacteria. **a**, Bubble plot showing metabolic potential of the metagenome-assembled genomes constructed from the three study sites (110 MAGs) and previously reported for the *Tara* Oceans dataset (1,877 MAGs). MAGs are summarized at phylum level, with the size of the circle corresponding to the number of genomes in that phylum with a given gene, and the colour reflecting the percentage of genome completeness. Marker genes that were not detected in any MAG are omitted. **b**, Maximum-likelihood phylogenetic tree of the catalytic subunit of the group 1 and 2 [NiFe]-hydrogenases. Hydrogenase sequences retrieved from the new MAGs (coloured green) and *Tara* MAGs (coloured blue) are shown alongside representative reference sequences (coloured yellow), including the three cultured marine bacteria (names in red). Evolutionary history was inferred by using the JTT matrix-based model, the tree was bootstrapped using 50 replicates and the tree is rooted using the outgroup group 4a [NiFe]-hydrogenase sequence. The tree includes hydrogenase subgroups implicated in aerobic respiration (groups 1d, 1f, 1l, 2a), anaerobic respiration (groups 1a, 1b, 1c, 1e) and H_2_ sensing (groups 2b and 2c). Source data

We subsequently determined the distribution of the metabolic marker genes in 110 metagenome-assembled genomes (MAGs) constructed from the local dataset and 1,888 previously reported MAGs (Fig. 3a and Supplementary Fig. 6) from the *Tara* Oceans dataset (Fig. 3a). The three lineages of aerobic H_2_-uptake hydrogenases were phylogenetically widespread, encoded by 75 (4.0%) of the bacterial MAGs, spanning 9 phyla and 26 orders, whereas CO dehydrogenases had a somewhat narrower distribution, that is, 70 (3.5%) MAGs, 6 phyla and 14 orders (Supplementary Table 5). Aerobic H_2_-uptake hydrogenases and CO dehydrogenases were both encoded by MAGs within the Proteobacteria, Bacteroidota, Actinobacteriota, Chloroflexota, Myxococcota and candidate phylum SAR324, and hydrogenases were also present in MAGs from the Cyanobacteria, Planctomycetota and Eremiobacterota (Fig. 3a). Phylogenetic trees depict the evolutionary history and taxonomic distributions of the catalytic subunits of the H_2_-oxidizing group 1 and 2 [NiFe]-hydrogenases (Fig. 3b and Supplementary Fig. 7), bidirectional group 3 and 4 [NiFe]-hydrogenases (Supplementary Fig. 8) and CO dehydrogenase (Supplementary Fig. 9).

Integrating genomic information with the wider literature, it is likely that H_2_ and CO oxidation support a myriad of lifestyles in marine ecosystems. The group 1d [NiFe]-hydrogenase was typically co-encoded with both ribulose 1,5-bisphosphate carboxylase/oxygenase (RuBisCO) and the sensory group 2b [NiFe]-hydrogenase in the MAGs of multiple Rhodobacteraceae, Alteromonadaceae and other Proteobacteria (Fig. 3b and Supplementary Table 5); this suggests that this enzyme supports hydrogenotrophic growth in H_2_-enriched waters, in line with the previously described roles of these hydrogenases in culture-based studies^11,38,51^. The group 1l [NiFe]-hydrogenase, recently shown to support persistence of a Bacteroidota isolate from Antarctic saline soils^4^, was encoded by predicted organoheterotrophs from the Bacteroidota, SAR324 and, on the basis of cultured isolates, Proteobacteria (Fig. 3b and Supplementary Table 5). Group 2a [NiFe]-hydrogenases, known to support mixotrophic growth of diverse bacteria^9^, were more phylogenetically diverse and taxonomically widespread; they were distributed in the MAGs of both predicted chemoorganoheterotrophs (Bacteroidota, Myxococcota, Proteobacteria) and photolithoautotrophs (Cyanobacteria) (Fig. 3b and Supplementary Table 5). CO dehydrogenases were mostly affiliated with Rhodobacteraceae and were also encoded by multiple MAGs from the classes Nanopelagicales S36-B12, Puniceispirillaceae, SAR324 NAC60-12 and Ilumatobacteraceae (Supplementary Fig. 9). Of *coxL*-encoding MAGs, 63% also encoded the genes for energy-converting rhodopsins or photosystem II, indicating that they can harvest energy concurrently or alternately from both CO and light, in support of previous culture-based findings^37^. While most of these MAGs are predicted to be obligate heterotrophs, 7% also encoded RuBisCO and hence are theoretically capable of carboxydotrophic growth (Supplementary Table 5). These findings support previous inferences that habitat generalists in marine waters benefit from metabolic flexibility, including consuming dissolved CO as a supplemental energy source^31,52^.

### H_2_ could support growth and survival of marine bacteria

**Fig. 4:**
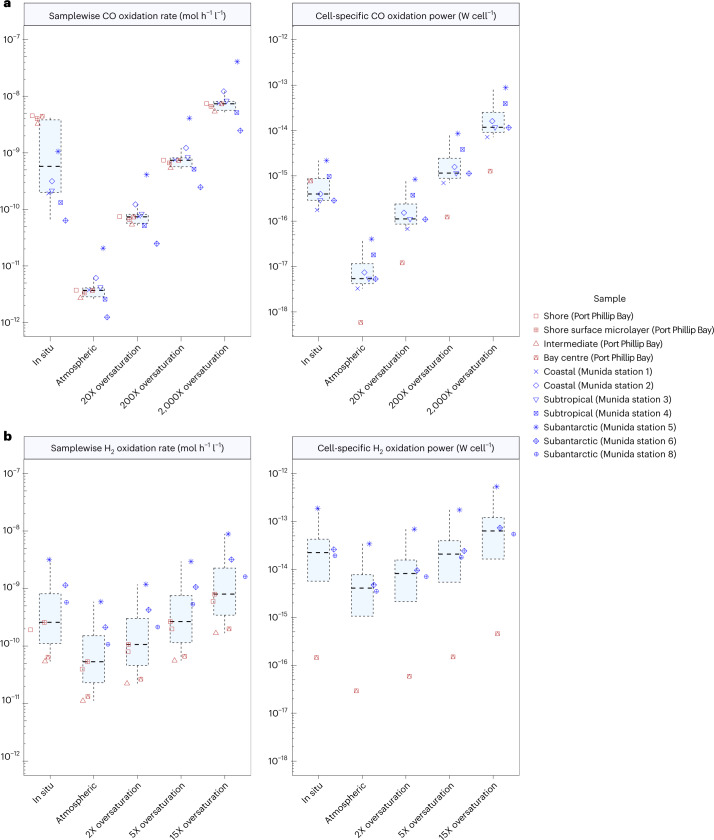
Thermodynamic modelling of H_2_ and CO oxidation by marine bacteria. **a**,**b**, The results show the bulk oxidation rates (left) and power yields per cell (right) for CO oxidation (**a**) (*n* = 10 (rate) and *n* = 7 (power) biologically independent samples) and H_2_ oxidation (**b**) (*n* = 7 (rate) and *n* = 4 (power) biologically independent samples). This analysis was only performed for samples where trace gas oxidation was measurable and cell-specific power was only calculated for samples where prokaryotic cell counts are available. Rates and power are shown on the basis of CO and H_2_ concentrations at a range of environmentally relevant concentrations. Centre values show medians, boxes show upper and lower quartiles, and whiskers show maximum (upper quartile plus 1.5 times interquartile range) and minimum (lower quartile minus 1.5 times interquartile range) values. Source data

We used two thermodynamic modelling calculations to estimate to what extent the measured rates of H_2_ and CO oxidation sustain cellular growth or survival. First, assuming a median maintenance energy of 1.9 × 10^−15^ Watts (W) per cell based on measurements of mostly copiotrophic isolates^53^, the measured oxidation rates would theoretically sustain an average of 2.0 × 10^7^ H_2_-oxidizing cells (range 1.4 × 10^6^ to 8.3 × 10^7^) and 6.1 × 10^7^ CO-oxidizing cells (range 2.1 × 10^6^ to 1.5 × 10^8^) per litre at in situ dissolved gas concentrations (Supplementary Table 1). Second, we calculated the amount of power (that is, W per cell) generated on the basis of the observed rates of trace gas oxidation (Fig. 1 and Supplementary Table 1) and predicted number of trace gas oxidizers (Fig. 2 and Supplementary Table 1) in the sampled waters, with this analysis being limited to the samples where oxidation was observed and reliable cell counts are available. On average, oxidation of the measured in situ concentrations of CO and H_2_ yields 7.2 × 10^−16^ W and 5.8 × 10^−14^ W per cell (Fig. 4). Together, these analyses suggest that the rates of CO oxidation are sufficient to sustain the survival, but not growth, of the numerous bacteria predicted to be capable of using this gas; this supports previous inferences that CO dehydrogenase primarily supports persistence in organoheterotrophic bacteria^6^.

In contrast, marine H_2_ oxidizers gain much power by oxidizing a relatively exclusive substrate at rapid cell-specific rates probably sufficient to support growth. The cell-specific power generated for the sample with the most active H_2_ oxidizers (5.4 × 10^−13^ W; from the first subantarctic station) is within the range reported for cellular metabolic rates of bacterial isolates during growth (median: 2.6 × 10^−14^ W; range: 2.8 × 10^−17^ to 2.1 × 10^−11^ W), and is higher than that of copiotrophic marine isolates *Vibrio* sp. DW1 (3.2 × 10^−14^ W) and *V. anguillarum* (1.8 × 10^−13^ W)^53^. While estimation of cell-specific power from community data is less precise than estimates derived from axenic culture, these power per cell calculations are probably underestimates, given that they do not account for any internal cycling of trace gases, assume that all cells are equally active and do not consider relic DNA. It should also be noted that the power gained per cell will substantially increase when H_2_ and CO become transiently highly elevated over space and time as depicted in Fig. 4. In combination with the genomic inferences that multiple MAGs encode hydrogenases known to support lithoautotrophic and lithoheterotrophic growth (Fig. 3), such thermodynamic modelling strongly suggests that a small proportion of bacteria in oceans can grow using H_2_ as an electron donor for aerobic respiration and, in some cases, CO_2_ fixation. By predominantly relying on energy derived from H_2_ oxidation, marine bacteria could potentially allocate most organic carbon for biosynthesis rather than respiration, that is, adopting a predominantly lithoheterotrophic lifestyle.

### A marine isolate uses atmospheric H_2_ mixotrophically

**Fig. 5:**
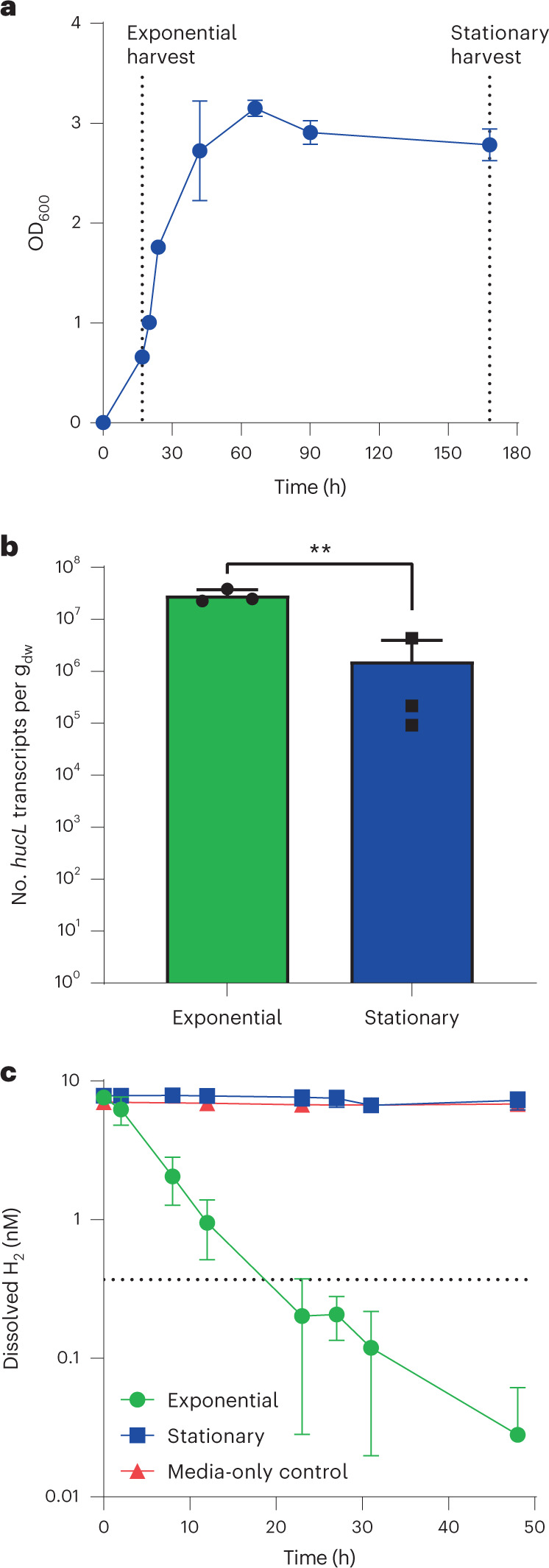
Hydrogenase expression and activity of *Sphingopyxis alaskensis*. **a**, Growth curve of *S. alaskensis* grown on Difco 2216 Marine Broth. Cultures were tested for gas consumption and collected for RT–qPCR in exponential phase (17 h, OD_600_ = 0.66) and stationary phase (168 h, 4 d post OD_max_). Data are presented as mean ± s.d., *n* = 3 biologically independent samples. **b**, Number of transcripts of the group 2a [NiFe]-hydrogenase large subunit gene (*hucL*; locus Sala_3198) as measured by RT–qPCR in exponential and stationary phase cultures of *S. alaskensis*. Mean ± s.d. of three biological replicates (averaged from two technical duplicates) per condition. The comparison is statistically significant based on an unpaired two-sided *t*-test (**P = 0.0062). **c**, H_2_ oxidation by exponential and stationary phase cultures of *S. alaskensis*. Mean ± s.d. of three biological replicates, with media-only vials monitored as negative controls. Dotted line shows the atmospheric concentration of hydrogen (0.53 ppmv). Source data

To better understand the mediators and roles of marine H_2_ oxidation, we investigated H_2_ uptake by three heterotrophic marine isolates encoding uptake hydrogenases closely related to those in the MAGs (Fig. 3). Two strains, *Robiginitalea biformata* DSM-15991 (Flavobacteriaceae)^54^ and *Marinovum algicola* FF3 (Rhodobacteraceae)^55^, did not substantially consume H_2_ over a 3 week period across a range of conditions despite encoding group 1l [NiFe]-hydrogenases. It is unclear whether hydrogenases have become non-functional in these fast-growing laboratory-adapted isolates or whether they are instead only active under very specific conditions. *Sphingopyxis alaskensis* RB2256 (Sphingomonadaceae)^56,57^, which encodes a plasmid-borne group 2a [NiFe]-hydrogenase, aerobically consumed H_2_ in a first-order kinetic process to sub-atmospheric levels (Fig. 5). Abundant in oligotrophic polar waters, *S. alaskensis* requires minimal resources to replicate since it forms extremely small cells (<0.1 µm^3^) and has a streamlined genome^57–60^. Previously thought to be an obligate organoheterotroph^61^, the discovery that this oligotrophic, exceptionally small bacterium (ultramicrobacterium)^62^ uses an abundant reduced gas as an energy source further rationalizes its ecological success. This is presumably the first report of atmospheric H_2_ oxidation by a marine bacterium.

We then determined whether *S. alaskensis* uses H_2_ oxidation primarily to support mixotrophic growth or survival. Expression of its hydrogenase large subunit gene (*hucL*) was quantified by reverse transcription quantitative PCR (RT–qPCR). Under ambient conditions, this gene was expressed at significantly higher levels (*P* = 0.006) during aerobic growth on organic carbon sources (mid-exponential phase; av. 2.9 × 10^7^ copies per g_dw_) than during survival (4 d in stationary phase; av. 1.5 × 10^6^ copies per g_dw_; *P* = 0.006) (Fig. 5a,b). This expression pattern is similar to other organisms possessing a group 2a [NiFe]-hydrogenase^9^ and is antithetical to that of the groups 1h and 1l [NiFe]-hydrogenases that are typically induced by starvation^1^. The activity of the hydrogenase was monitored under the same two conditions by monitoring depletion of headspace H_2_ mixing ratios over time by gas chromatography. H_2_ was rapidly oxidized by exponentially growing cultures to sub-atmospheric concentrations within a period of 30 h, whereas negligible consumption occurred in stationary phase cultures (Fig. 5c). Together, these findings suggest that *S. alaskensis* can grow mixotrophically in marine waters by simultaneously consuming dissolved H_2_ with available organic substrates. These findings align closely with that observed for other organisms harbouring group 2a [NiFe]-hydrogenases^9,10^ and support the inferences from thermodynamic modelling (Fig. 4) that H_2_ probably supports growth of some marine bacteria.

### H_2_ and CO oxidation capacity changes with water depth

**Fig. 6:**
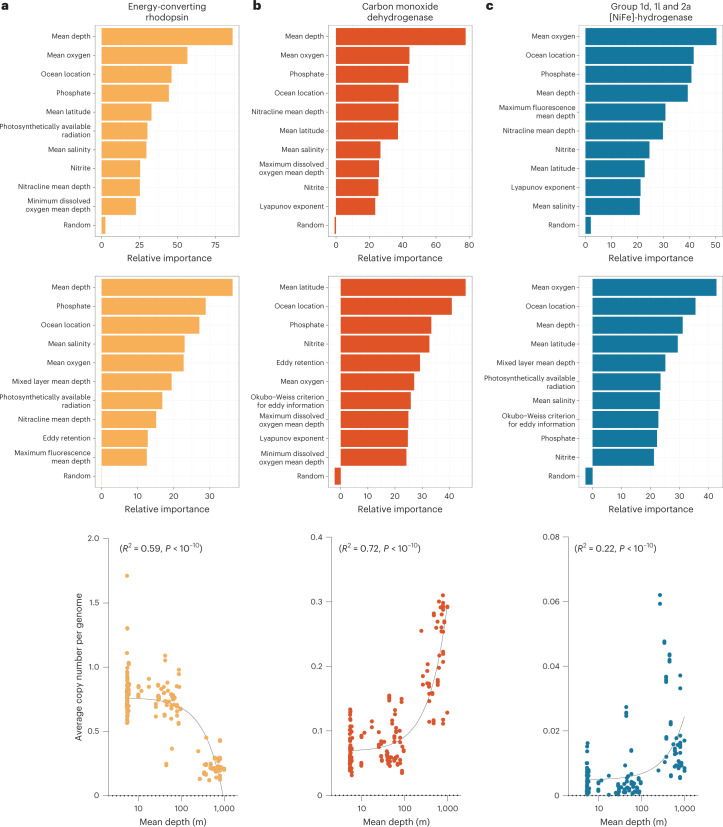
Drivers of the abundance and expression of metabolic genes in *Tara* Oceans metagenomes. **a**–**c**, This analysis is visualized for energy-converting rhodopsins (**a**), CO dehydrogenases (**b**) and aerobic H_2_-uptake hydrogenases (**c**). The top and middle panels show random forest modelling of the environmental variables that best predict marker gene abundance in metagenomes and metatranscriptomes, respectively. The relative importance (percentage increase in mean squared error, %IncMSE, as a measure of decrease in model accuracy) of the top ten most important variables for each model is shown in addition to a randomized variable used to benchmark importance. The bottom panel shows simple linear correlations between the metagenomic abundance of each gene and water depth. For each gene, Pearson’s *R*^2^ values show goodness of fit and *P* values confirm that each slope significantly deviates from zero. Source data

Finally, we investigated the environmental correlates of the abundance and expression of trace gas oxidation genes in the *Tara* Oceans datasets (Fig. 6 and Supplementary Table 6). Linear correlation analysis confirmed that genes encoding the aerobic H_2_-uptake hydrogenase (*R*^2^ = 0.22, *P* < 0.0001) and the CO dehydrogenase (*R*^2^ = 0.72, *P* < 0.0001) both significantly increased with depth (Fig. 6), as illustrated by their increased abundance in the metagenomes from mesopelagic waters (Fig. 2). This contrasts with the sharp decreases in the genes responsible for phototrophy, such as energy-converting rhodopsins (*R*^2^ = 0.59, *P* < 0.0001), with depth (Figs. 2 and 6). This pattern was consistent across sites in the Atlantic, Indian, Pacific and Southern Oceans. These findings suggest that as light and hence energy availability decreases, there is a greater selective advantage for bacteria that use trace gases (lithoheterotrophy) rather than photosynthesis (photoheterotrophy).

These inferences were nuanced, after accounting for co-correlated variables (Supplementary Fig. 10), by random forest modelling (Fig. 6 and Supplementary Figs. 11 and 12). Depth was among the top three strongest predictors of the abundance of group 1l and 2a [NiFe]-hydrogenases, CO dehydrogenase and energy-converting rhodopsins (Fig. 6 and Supplementary Fig. 11). Latitude proved to be a strong predictor of the expression of the group 1l [NiFe]-hydrogenases and CO dehydrogenases, the latter peaking in the tropics (Fig. 6 and Supplementary Figs. 11–13). One explanation for the latter is that in tropical waters, increased photochemical and thermochemical CO production enhances substrate availability for CO oxidizers. These observations are consistent with the inverse CO and H_2_ oxidation rates observed across the Munida transect (Fig. 1), as well as previously reported latitudinal variations in seawater concentrations of these gases^23–28^. In contrast, group 1d [NiFe]-hydrogenase gene abundance and expression levels were highest in hypoxic waters (Fig. 6 and Supplementary Fig. 14); this suggests that in contrast to its high-affinity oxygen-insensitive counterparts, this hydrogenase will be most transcribed when H_2_ levels are elevated due to hypoxic fermentation (resulting in activation of the sensory hydrogenase) and most active when O_2_ levels are low enough to minimize active site inhibition^38,51^. Collectively, our analyses suggest that there are complex environmental controls on the abundance and activities of marine trace gas oxidizers, and that the three H_2_-uptake hydrogenases are ecophysiologically distinct.

## Discussion

Through an integrative approach, we provide presumably the first demonstration that H_2_ is an important energy source for seawater communities. The biogeochemical, metagenomic and thermodynamic modelling analyses together suggest that H_2_ is oxidized by a diverse but small proportion of community members, but at sufficiently fast cell-specific rates to enable lithotrophic growth. These findings are supported by experimental observations that the ultramicrobacterium *S. alaskensis* consumes H_2_ during heterotrophic growth. Marine bacteria with the capacity to oxidize H_2_ probably gain a major competitive advantage from being able to consume this abundant, diffusible, high-energy gas. H_2_-oxidizing marine microorganisms are globally distributed, although activity measurements and hydrogenase distribution profiles suggest complex controls on their activity and that they may be particularly active in low-chlorophyll waters. In contrast, our findings support that CO oxidation is a widespread trait that enhances the flexibility and likely primarily survival of habitat generalists^30,31^, especially in high-chlorophyll waters. At the biogeochemical scale, our findings indicate that marine bacteria mitigate atmospheric H_2_ emissions^19^ and potentially account for undersaturation of H_2_ in Antarctic waters^28^.

Yet a major enigma remains. H_2_ and CO are among the most dependable energy sources in the sea given their relatively high concentrations and energy yields. So why do relatively few bacteria harness them? By comparison, soils are net sinks for these trace gases given that the numerous bacteria present rapidly consume them^12^. We propose the straightforward explanation that the resource investment required to make the metalloenzymes to harness these trace gases may not always be justified by the energy gained. In the acutely iron-limited ocean, hydrogenases (containing 12–13 Fe atoms per protomer^11^) and to a lesser extent CO dehydrogenases (containing 4 Fe atoms per protomer^63^) are a major investment. This trade-off is likely to be most pronounced in the surface ocean, where solar energy can be harvested using minimal resources through energy-converting rhodopsins. However, the iron investment required to consume H_2_ and CO is likely to be justified in energy-limited waters at depths and regions or seasons where primary production is low. This is consistent with the observed enrichment of hydrogenases and CO dehydrogenases in metagenomes from mesopelagic waters, as well as increased H_2_ oxidation observed in subantarctic waters. Moreover, iron availability is typically higher in deeper circulating waters and around continental shelves (due to both deep water upwelling and terrestrial inputs), where high hydrogenase expression and activity were observed^64^. Thus, oceans continue to be a net source of H_2_ and CO despite the importance of these energy sources for diverse marine bacteria.

## Methods

### Sample collection and characteristics

To determine the ability of marine microbial communities to oxidize trace gases, a total of 14 marine surface water samples were collected from three different locations (Supplementary Fig. 1). Eight samples were collected from across the Munida Microbial Observatory Time-Series transect (Otago, New Zealand)^65^ on 23 July 2019 in calm weather on the RV *Polaris II*. This marine transect begins off the coast of Otago, New Zealand and extends through neritic, subtropical and subantarctic waters^65^. Eight equidistant stations were sampled travelling east, ranging from approximately 15 km to 70 km from Taiaroa Head. At each station, water was collected at 1 m depth using Niskin bottles and stored in two 1 l autoclaved bottles. One bottle was reserved for DNA filtration and extraction, whereas the other was used for microcosm incubation experiments. The vessel measured changes in salinity and temperature to determine the boundaries of each water mass (Supplementary Fig. 2).

Four samples were also collected from the temperate Port Phillip Bay at Carrum Beach (Victoria, Australia) on 20 March 2019 and two were collected from the tropical Heron Island (Queensland, Australia) on 9 July 2019. At both sites, near-shore surface microlayer and surface water samples were collected in the subtidal zone (water depth ca. 1 m). At Port Phillip Bay, two samples were also collected at 7.5 km and 15 km east of the mouth of the Patterson River, labelled ‘Intermediate’ and ‘Centre’ respectively. In all cases, surface water samples of 3 l were collected with a sterile Schott bottle from approximately 20 cm depth and aliquoted for microcosm incubation and DNA extraction. Surface microlayer samples were collected using a manual glass-plate sampler of 1,800 cm^2^ surface area^66^. A total of 520–580 ml was collected in 150–155 dips, resulting in an average sampling thickness of 20 µm. For the surface microlayer samples, 180 ml was reserved for microcosm incubations, with the remaining volume used for DNA extraction. From all transects, each sample reserved for DNA extraction was vacuum-filtered using 0.22 µm polycarbonate filters and then stored at −80 °C until extraction.

### Measurement of dissolved H_2_ and CO

Dissolved gases were also sampled in situ at each transect to measure dissolved concentrations of CO and H_2_. Serum vials (160 ml) were filled with seawater using a gas-tight tube, allowing approximately 300 ml to overflow. The vial was then sealed with a treated lab-grade butyl rubber stopper, avoiding the introduction of gas to the vial. An ultra-pure N_2_ headspace (20 ml) was introduced to the vial by concurrently removing 20 ml of liquid using two gas-tight syringes. The vials were then shaken vigorously for 2 min before being equilibrated for 5 min to allow dissolved gases to enter the headspace. Of the headspace, 17 ml was then collected into a syringe flushed with N_2_ by returning the removed liquid to the vial, and 2 ml was purged to flush the stopcock and needle before injecting the remaining 15 ml into a N_2_-flushed and evacuated silicone-closed Exetainer^67^ for storage. Exetainers were sealed with a stainless-steel bolt and O-ring and stored until measurement. H_2_ and CO concentrations in the Exetainers were analysed by gas chromatography using a pulse discharge helium ionization detector (model TGA-6792-W-4U-2, Valco Instruments), as previously described^68^, calibrated against standard CO and H_2_ gas mixtures of known concentrations.

### Ex situ activity assays

To determine the ability of these marine microbial communities to oxidize CO and H_2_, the seawater samples were incubated with these gases under laboratory conditions and their concentration over time was measured using gas chromatography. For each sample, triplicate microcosms were setup in which seawater was transferred into foil-wrapped serum vials (60 ml seawater in 120 ml vials for Munida transect and Port Phillip Bay; 80 ml seawater in 160 ml vials for Heron Island) and sealed with treated lab-grade butyl rubber stoppers^67^. For each sampling location, one set of triplicates was also autoclaved and used as a control. The ambient-air headspace of each vial was spiked with H_2_ and CO so that they reached initial headspace mixing ratios of either 2 ppmv (Munida transect and Port Phillip Bay) or 10 ppmv (Heron Island). Microcosms were continuously agitated at 20 °C on a shaker table at 100 r.p.m. For Munida and Port Phillip Bay samples, 1 ml samples were extracted daily from the headspace and their content was measured by gas chromatography as described above. For Heron Island samples, at each timepoint, 6 ml gas was extracted and stored in 12 ml UHP-He-flushed conventional Exetainers (2018) or pre-evacuated 3 ml silicone-sealed Exetainers^67^.

### Calculation of dissolved gas concentrations

The concentrations of dissolved gases in seawater at equilibrium state and at 1 atmospheric pressure were calculated according to the Sechenov relation for mixed electrolyte solutions, as described in ref. ^69^:

1 $$\log \left( {\frac{{k_{G,0}}}{{k_G}}} \right) = {\sum} {\left( {h_i + h_G} \right)c_i}$$

where *k*_*G*,0_ and *k*_*G*_ denote the gas solubility (or Henry’s law constant in equivalent) in water and the mixed electrolyte solution, respectively, *h*_*i*_ is a constant specific to the dissolved ion *i* (m^3^ kmol^−1^), *h*_*G*_ is a gas-specific parameter (m^3^ kmol^−1^) and *c*_*i*_ represents the concentration of the dissolved ion *i* in solution (kmol m^−3^). The gas-specific constant, *h*_*G*_, at temperature *T* (in K) follows the equation:

2 $$h_G = h_{G,0} + h_T\left( {T - 298.15} \right)$$

where *h*_*G*,0_ represents the value of *h*_*G*_ at 298.15 K and *h*_*T*_ is a gas-specific parameter for the temperature effect (m^3^ kmol^−1^ K^−1^). The gas solubility parameter *k*_*G*,0_ at temperature *T* follows combined Henry’s law and van ’t Hoff equation:

3 $$k_{G,0} = k_{G,0}^\prime \times e^{\frac{{ - {\Delta}_{\mathrm{soln}}H}}{R}\left( {\frac{1}{T} - \frac{1}{{298.15}}} \right)}$$

where $k_{G,0}^\prime$ denotes Henry’s law constant of the gas at 298.15 K, ${\Delta}_{\mathrm{soln}}H$ is the enthalpy of solution and *R* is the ideal gas law constant.

The concentrations of dissolved gases at equilibrium with the headspace gas phase at 1 atmospheric pressure and incubation temperature of 20 °C were calculated on the basis of a mean seawater composition as reported in ref. ^70^. The salinity correcting constants *h*_*i*_, *h*_*G*,0_ and *h*_*T*_ were adopted from ref. ^69^, while the temperature correcting constants $k_{G,0}^\prime$ and ${\textstyle{{ - {\Delta}_{\mathrm{soln}}H} \over R}}$ were obtained from ref. ^71^.

### Kinetic analysis and thermodynamic modelling

For kinetic analysis, measurement timepoints of up to 30 d of incubation time were used. The gas consumption pattern was fitted with both an exponential model and a linear model. The former showed a lowest overall Akaike information criterion value for both H_2_ and CO consumption (Supplementary Table 1). As such, first-order reaction rate constants were calculated and used for the kinetic modelling. In addition, only samples having at least two replicates with a positive rate constant were deemed to have a confident gas consumption. Bulk atmospheric gas oxidation rates for each sample were calculated with respect to the mean atmospheric mixing ratio of the corresponding trace gases (H_2_: 0.53 ppmv; CO: 0.09 ppmv; CH_4_: 1.9 ppmv). To estimate the cell-specific gas oxidation rate, the average direct cell count values reported for surface seawaters at Port Phillip Bay centre^72^ and the eight stations along the Munida transect were used^65,73^. Assuming that all cells are viable and active, cell-specific gas oxidation rates were then inferred by multiplying the estimated relative abundance of trace gas oxidizers derived from the metagenomic short reads (the average gene copy number, assuming one copy per organism; see ‘Metabolic annotation’ below) by the cell counts to obtain the number of trace gas oxidizers.

To estimate the energetic contributions of H_2_ and CO oxidation to the corresponding marine trace gas oxidizers, we performed thermodynamic modelling to calculate their respective theoretical energy yields according to the first-order kinetics of each sample estimated above. Power (Gibbs energy per unit time per cell) *P* follows the equation:

4 $$P = \frac{{v \times {\Delta}G_{\mathrm{r}}}}{B}$$

where *v* denotes the rate of substrate consumption per litre of seawater (mol l^−1^ s^−1^) and *B* is the number of microbial cells (cells l^−1^) performing the reactions H_2_ + 0.5 O_2_ → H_2_O (dihydrogen oxidation) and CO + 0.5 O_2_ → CO_2_ (carbon monoxide oxidation). Δ*G*_r_ represents the Gibbs free energy of the reaction at the experimental conditions (J mol^−1^) and follows the equation:

5 $${{{\mathrm{{\Delta}}}}}G_{\mathrm{r}} = {{{\mathrm{{\Delta}}}}}G_{\mathrm{r}}^0 + RT\,{{{\mathrm{ln}}}}Q_{\mathrm{r}}$$

where ${\Delta}G_{\mathrm{r}}^0$ denotes the standard Gibbs free energy of the reaction, *Q*_r_ denotes the reaction quotient, *R* represents the ideal gas constant and *T* represents temperature in Kelvin. Values of ${\Delta}G_{\mathrm{r}}^0$ of the hydrogen oxidation and carbon monoxide oxidation were obtained from ref. ^74^. Values of *Q*_r_ for each reaction were calculated using:

6 $$Q_{\mathrm{r}} = {\prod} {a_g^{n_i}}$$

where *a*_*g*_ and *n*_i_ denote the dissolved concentration of the *i*th species in seawater and the stoichiometric coefficient of the *i*th species in the reaction of interest, respectively. Gibbs free energies were calculated for oxidation of hydrogen and carbon monoxide at atmospheric pressure and 20 °C incubation temperature. To contextualize cellular power yield from H_2_ and CO oxidation in relation to reported cellular energy requirements, a comprehensive list of maintenance (endogenous rate) and growth (active rate) power requirements of 121 organoheterotrophic bacteria at 20 °C reported in ref. ^53^ was used as the primary reference. A median maintenance energy of 1.9 × 10^−15^ W per cell was derived from the bacterial endogenous rates obtained in the supporting information sd01 of the above reference.

### Metagenomic sequencing and assembly

DNA was extracted from the sample filters using the DNeasy PowerSoil kit (QIAGEN) following the manufacturer’s instructions. Sample libraries, including an extraction blank control, were prepared with the Nextera XT DNA Sample Preparation kit (Illumina) and sequenced on an Illumina NextSeq500 platform (2 × 151 bp) at the Australian Centre for Ecogenomics (University of Queensland). An average of 20,122,526 read pairs were generated per sample, with 827,868 read pairs sequenced in the negative control (Supplementary Table 2). Raw metagenomic data were quality controlled with the BBTools suite v38.90 (https://sourceforge.net/projects/bbmap/), using BBDuk to remove the 151st base, trim adapters, filter PhiX reads, trim the 3’ end at a quality threshold of 15 and discard reads below 50 bp in length. Reads detected in the extraction blank were additionally removed with BBMap v38.90, leaving a total of 97.7% of raw sample reads for further analysis. Taxonomy was profiled from high-quality short reads by assembling and classifying 16S rRNA and 18S rRNA genes with PhyloFlash v3.4 (ref. ^75^). Short reads were assembled individually with metaSPAdes v3.14.1 (ref. ^76^) and collectively (all samples together, and by location) with MEGAHIT v1.2.9 (ref. ^77^). Coverage profiles for each contig were generated by mapping the short reads to the assemblies with BBMap v38.90 (ref. ^78^).

Genome binning was performed with MetaBAT2 v2.15.5 (ref. ^79^), MaxBin 2 v2.2.7 (ref. ^80^) and CONCOCT v1.1.0 (ref. ^81^) after setting each tool to retain only contigs ≥2,000 bp in length. For each assembly, resulting bins were dereplicated across binning tools with DAS_Tool v1.1.3 (ref. ^82^). All bins were refined with RefineM v0.1.2 (ref. ^83^) and consolidated into a final set of non-redundant metagenome-assembled-genomes (MAGs) at the default 99% average nucleotide identity using dRep v3.2.2 (ref. ^84^). The completeness, contamination and strain heterogeneity of each MAG were calculated with CheckM v1.1.3 (ref. ^85^), resulting in a total of 21 high-quality (>90% completeness, <5% contamination^86^) and 89 medium-quality (>50% completeness, <10% contamination^86^) MAGs. Taxonomy was assigned to each MAG with GTDB-Tk v1.6.0 (ref. ^87^) (using GTDB release 202)^88^ and open reading frames were predicted from each MAG and additionally across all contigs (binned and unbinned) with Prodigal v2.6.3 (ref. ^89^). CoverM v0.6.1 (https://github.com/wwood/CoverM) ‘genome’ was used to calculate the relative abundance of each MAG in each sample (–min-read-aligned-percent 0.75,–min-read-percent-identity 0.95,–min-covered-fraction 0) and the mean read coverage per MAG across the dataset (-m mean,–min-covered-fraction 0).

For global comparisons, raw metagenome (PRJEB1787) and metatranscriptome (PRJEB6608) data from the *Tara* Oceans global dataset were downloaded from the European Nucleotide Archive^47,50^. In addition, 1,888 bacterial and archaeal MAGs generated in ref. ^90^ were downloaded (via https://www.genoscope.cns.fr/tara/).

### Metabolic annotation

For both the metagenomes generated in this study and those from the *Tara* Oceans dataset, high-quality short reads and predicted proteins from assemblies and MAGs underwent metabolic annotation using DIAMOND v2.0.9 (–max-target-seqs 1,–max-hsps 1)^91^ for alignment against a custom set of 50 metabolic marker protein databases. The marker proteins (10.26180/c.5230745) cover the major pathways for aerobic and anaerobic respiration, energy conservation from organic and inorganic compounds, carbon fixation, nitrogen fixation and phototrophy^4^. Gene hits were filtered as follows: alignments were filtered to retain only those either at least 40 amino acids in length (150 bp metagenomes from the current study), 32 amino acids in length (100 bp *Tara* metagenomes and metatranscriptomes) or with at least 80% query or 80% subject coverage (predicted proteins from assemblies and MAGs). Alignments were further filtered by a minimum percentage identity score by protein: for short reads, this was 80% (PsaA), 75% (HbsT), 70% (PsbA, IsoA, AtpA, YgfK and ARO), 60% (CoxL, MmoA, AmoA, NxrA, RbcL, NuoF, FeFe hydrogenases and NiFe Group 4 hydrogenases) or 50% (all other genes). For predicted proteins, the same thresholds were used except for AtpA (60%), PsbA (60%), RdhA (45%), Cyc2 (35%) and RHO (30%).

For short reads, gene abundance in the community was estimated as ‘average gene copies per organism’ by dividing the abundance of the gene (in reads per kilobase million, RPKM) by the mean abundance of 14 universal single-copy ribosomal marker genes (in RPKM, obtained from the SingleM v0.13.2 package, https://github.com/wwood/singlem). For single-copy metabolic genes, this corresponds to the proportion of community members that encode the gene. A linear correlation analysis, performed in GraphPad Prism 9, was used to determine how metagenomic gene abundance correlated with ex situ H_2_ and CO oxidation rates. For the *Tara* Oceans dataset, the RNA:DNA ratio was calculated by dividing gene abundance in the metatranscriptome (in RPKM) by the gene abundance in the corresponding metagenome (RPKM) to examine gene expression relative to abundance. Where replicate metagenomes or metatranscriptomes were present, RPKM values were averaged by sample.

### Phylogenetic analysis

Phylogenetic trees were constructed to understand the distribution and diversity of marine microorganisms capable of H_2_ and CO oxidation. Trees were constructed for the catalytic subunits of the groups 1 and 2 [NiFe]-hydrogenases, groups 3 and 4 [NiFe]-hydrogenases, and the form I CO dehydrogenase (CoxL). In all cases, protein sequences retrieved from the MAGs by homology-based searches were aligned against a subset of reference sequences from custom protein databases^6,49^ using ClustalW in MEGA11 (ref. ^92^). In brief, evolutionary relationships were visualized by constructing a maximum-likelihood phylogenetic tree; specifically, initial trees for the heuristic search were obtained automatically by applying Neighbour-Join and BioNJ algorithms to a matrix of pairwise distances estimated using a Jones-Taylor-Thornton (JTT) model, and then selecting the topology with superior log likelihood value within MEGA11. All residues were used and trees were bootstrapped with 50 replicates. Phylogenetic tree annotation and visualization were performed using iTOL (v6.6).

### Environmental driver analysis

Random forest models, Pearson correlations and Spearman correlations were generated for the *Tara* Oceans dataset to identify significant correlations between sample environmental metadata and the normalized abundance of carbon monoxide dehydrogenase, rhodopsin and [NiFe] groups 1d, 1e, 1l, 2a, 3b and 3d hydrogenase genes (shown as copies per organism for metagenomes, log_10_(RPKM + 1) for metatranscriptomes). To account for collinearity, where environmental variables were highly correlated (Pearson coefficient > |0.7|, Supplementary Fig. 10), one was excluded from the random forest models to avoid the division of variable importance across those features. These excluded variables were selected at random, unless they were highly correlated with depth (which was kept). Then, using imputed values where data were missing (function rfImpute()), a random forest model was generated for each gene above using the environmental variables marked in Supplementary Table 6 as predictors (importance = TRUE, ntree = 3,000), using the R package randomForest^93^. All combinations of the above genes and environmental variables were additionally correlated with Pearson’s and Spearman’s rank correlations, omitting missing values and adjusting all *P* values with the false discovery rate correction.

### Culture-based growth and gas consumption analysis

Axenic cultures of three bacterial strains were analysed in this study: *Sphingopyxis alaskensis* (RB2256)^56,57^ obtained from UNSW Sydney, *Robiginitalea biformata* DSM-15991 (ref. ^54^) imported from DSMZ and *Marinovum algicola* FF3 (Rhodobacteraceae)^55^ imported from DSMZ. Cultures were maintained in 120 ml glass serum vials containing a headspace of ambient air (H_2_ mixing ratio ~0.5 ppmv) sealed with treated lab-grade butyl rubber stoppers^67^. Broth cultures of all three species were grown in 30 ml of Difco 2216 Marine Broth media and incubated at 30 °C at an agitation speed of 150 r.p.m. in a Ratek orbital mixer incubator with access to natural day/night cycles. Growth was monitored by determining the optical density (OD_600_) of periodically sampled 1 ml extracts using an Eppendorf BioSpectrophotometer. The ability of the three cultures to oxidize H_2_ was measured by gas chromatography. Cultures in biological triplicate were opened, equilibrated with ambient air (1 h) and resealed. These re-aerated vials were then amended with H_2_ (via 1% v/v H_2_ in N_2_ gas cylinder, 99.999% pure) to achieve final headspace concentrations of ~10 ppmv. Headspace mixing ratios were measured immediately after closure and at regular intervals thereafter until the limit of quantification of the gas chromatograph was reached (42 ppbv H_2_). This analysis was performed for both exponential (OD_600_ 0.67 for *S. alaskensis*) and stationary phase cultures (~72 h post OD_max_ for *S. alaskensis*).

### RT–qPCR analysis

Quantitative reverse transcription PCR (RT–qPCR) was used to determine the expression levels of the group 2a [NiFe]-hydrogenase large subunit gene (*hucL*; locus Sala_3198) in *S. alaskensis* during growth and survival. For RNA extraction, triplicate 30 ml cultures of *S. alaskensis* were grown synchronously in 120 ml sealed serum vials. Cultures were grown to either exponential phase (OD_600_ 0.67) or stationary phase (48 h post OD_max_ ~3.2). Cells were then quenched using a glycerol-saline solution (−20 °C, 3:2 v/v), collected by centrifugation (20,000 × *g*, 30 min, −9 °C), resuspended in 1 ml cold 1:1 glycerol:saline solution (−20 °C) and further centrifuged (20,000 × *g*, 30 min, −9 °C). Briefly, resultant cell pellets were resuspended in 1 ml TRIzol reagent (Thermo Fisher), mixed with 0.1 mm zircon beads (0.3 g) and subjected to bead beating (three 30 s on/30 s off cycles, 5,000 r.p.m.) in a Precellys 24 homogenizer (Bertin Technologies) before centrifugation (12,000 × *g*, 10 min, 4 °C). Total RNA was extracted using the phenol-chloroform method following the manufacturer’s instructions (TRIzol reagent user guide, Thermo Fisher) and resuspended in diethylpyrocarbonate-treated water. RNA was treated using the TURBO DNA-free kit (Thermo Fisher) following the manufacturer’s instructions. RNA concentration and purity were confirmed using a NanoDrop ND-1000 spectrophotometer.

Complementary DNA was synthesized using a SuperScript III First-Strand Synthesis System kit for RT–qPCR (Thermo Fisher) with random hexamer primers, following the manufacturer’s instructions. RT–qPCR was performed in a QuantStudio 7 Flex Real-Time PCR System (Applied Biosystems) using a LightCycler 480 SYBR Green I Master Mix (Roche) in 96-well plates according to the manufacturer’s instructions. Primers were designed using Primer3 (ref. ^94^) to target the *hucL* gene (HucL_fw: AGCTACACAAACCCTCGACA; HucL_rvs: AGTCGATCATGAACAGGCCA) and the 16S rRNA gene as a housekeeping gene (16S_fwd: AACCCTCATCCCTAGTTGCC; 16S_rvs: GGTTAGAGCATTGCCTTCGG). Copy numbers for each gene were interpolated from standard curves of each gene created from threshold cycle (*C*_T_) values of amplicons that were serially diluted from 10^8^ to 10 copies (*R*^2^ > 0.98). Hydrogenase expression data were then normalized to the housekeeping gene in exponential phase. All biological triplicate samples, standards and negative controls were run in technical duplicate. A Student’s *t*-test in GraphPad Prism 9 was used to compare *hucL* expression levels between exponential and stationary phases.

### Reporting summary

Further information on research design is available in the Nature Portfolio Reporting Summary linked to this article.

## Supplementary information

Supplementary Information Supplementary Figs. 1–14 and legends for Supplementary Tables 1–6.

Reporting Summary

Supplementary Tables 1–6. Excel workbook containing Supplementary Tables 1–6, most of which comprise multiple tabs. The table number is designated in the first row of each spreadsheet, and also in the tab names.

## Data Availability

All raw metagenomes and metagenome-assembled genomes are deposited to the NCBI Sequence Read Archive under the BioProject accession number PRJNA801081. Raw metagenome (PRJEB1787) and metatranscriptome (PRJEB6608) data from the Tara Oceans global dataset were downloaded from the European Nucleotide Archive (https://www.ebi.ac.uk/ena/browser/view/PRJEB402). Bacterial and archaeal MAGs (1,888) generated in ref. ^90^ were downloaded from https://www.genoscope.cns.fr/tara/. The metabolic marker protein database used in this study, which includes reference hydrogenase and carbon monoxide dehydrogenase sequences, can be obtained from https://bridges.monash.edu/collections/_/5230745.

## Source data

Source Data Fig. 1 Source data for Fig. 1.

Source Data Fig. 2 Source data for Fig. 2.

Source Data Fig. 3 Source data for Fig. 3.

Source Data Fig. 4 Source data for Fig. 4.

Source Data Fig. 5 Source data for Fig. 5.

Source Data Fig. 6 Source data for Fig. 6.

## Code availability

Metagenomics analysis scripts are publicly available at https://github.com/greeninglab/MarineOxidationManuscript.
