# Supplementary material for: Molecular hydrogen in seawater supports growth of diverse marine bacteria
Source: Nat Microbiol. 2023 Feb 6;8(4):581–95. doi: 10.1038/s41564-023-01322-0 (PMC10305171; doi:10.1038/s41564-023-01322-0)

nature microbiology

Article https://doi.org/10.1038/s41564-023-01322-0

Molecular hydrogen in seawater supports growth of diverse marine bacteria

In the format provided by the authors and unedited

Supplementary information

Table S1 (xlsx). Trace gas oxidation rates and power calculations for Port Phillip Bay and Munida Transect samples.

Table S2 (xlsx). Sequencing statistics of the 15 newly sequenced metagenomes.

Table S3 (xlsx). Abundance of metabolic marker genes in the short read metagenomic and metatranscriptome data. For the newly sequenced metagenomes, this includes the calculated average copies per organism for each marker gene, average copies per organism for each hydrogenase subgroup, and a list of short-read hits and their corresponding match in the database. For the *Tara* Oceans dataset, this includes the calculated average copies per organism, transcript levels, and expression ratios (RNA:DNA ratio) for each marker gene and hydrogenase subgroup.

Table S4 (xlsx). Marker genes identified in the assembled contigs in the newly sequenced metagenomes. This lists the metabolic marker genes annotated across all contigs over 2000 bp (including both unbinned contigs and those that were assigned to a bin).

Table S5 (xlsx). Summary and annotation of metagenome-assembled-genomes (MAGs). For the newly sequenced metagenomes, this includes taxonomic information for each MAG, relative abundance of each MAG per sample calculated by CoverM for the newly sequenced genomes, a summary of metabolic marker genes identified in each MAG, and a full list of metabolic marker genes identified in the MAGs with

alignment information and protein sequences. For the *Tara* Oceans metagenomes, this includes taxonomic information and metabolic summary for each MAG.

Table S6 (xlsx). Spearman's correlation analyses based on metadata from the *Tara* Oceans metagenomes and metatranscriptomes. Raw and false discovery rate adjusted two-sided p-values are provided, and were calculated using the asymptotic *t* approximation (R function cor.test(), method = "spearman", exact = FALSE).

Figure S1. Map of the sites sampled in this study.

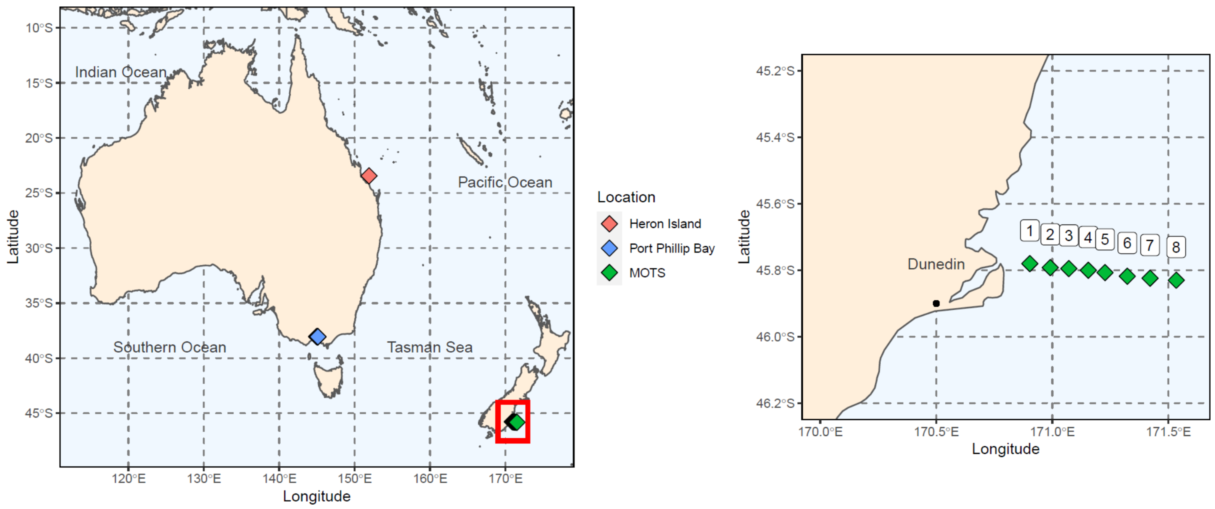

Figure S2. Eight stations sampled in the Munida Observation Time Series. The transect extends 65 km in an Eastern direction from Taieri Head, Otago. It spans neritic waters (NW; black; two stations), a transitional subtropical frontal zone (STW; yellow; two stations), and subantarctic waters (SAW; blue; four stations). Temperature and salinity data reflect the transitions between the three zones.

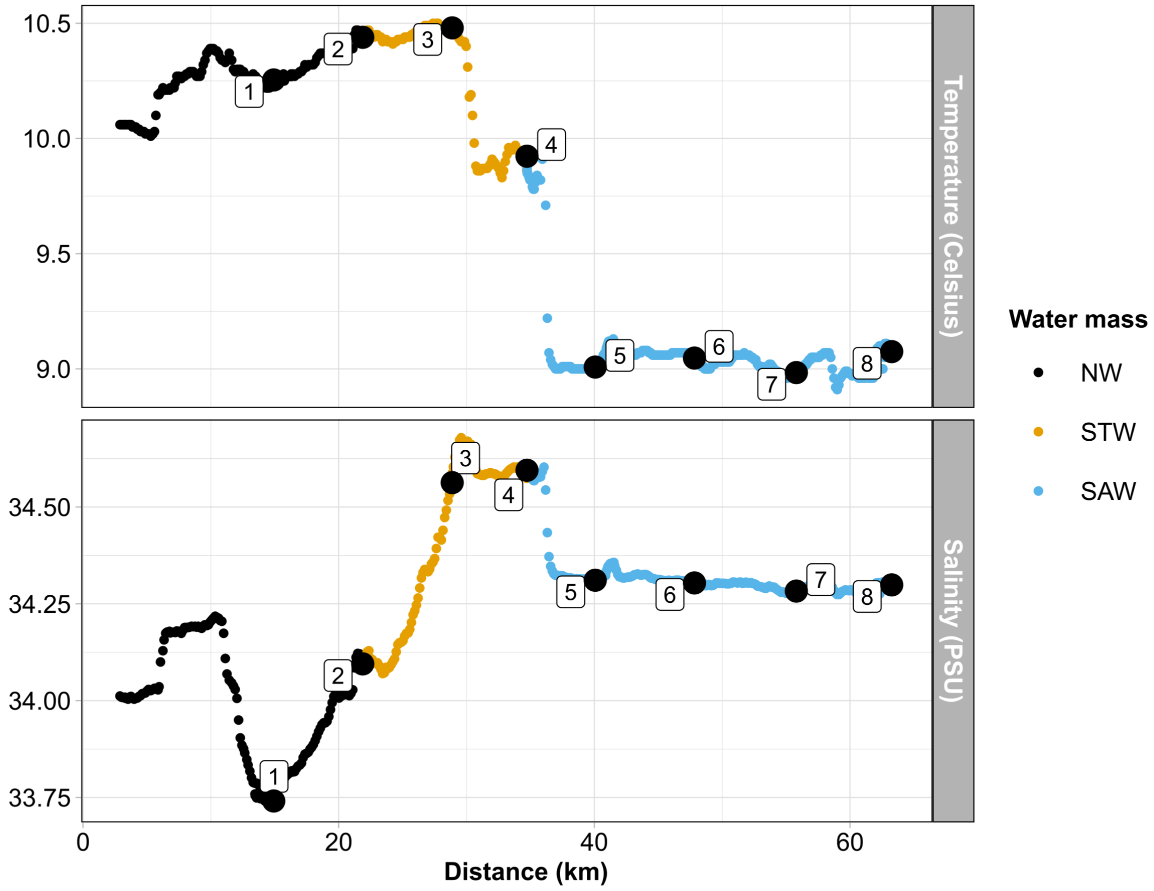

Figure S3. Trace gas oxidation by marine microbial communities in Heron Island. Samples were spiked with 10 ppm CO and H_2 and incubated in the dark. Relative concentrations were calculated by dividing concentrations at later times with the measured concentration at the start of each incubation.

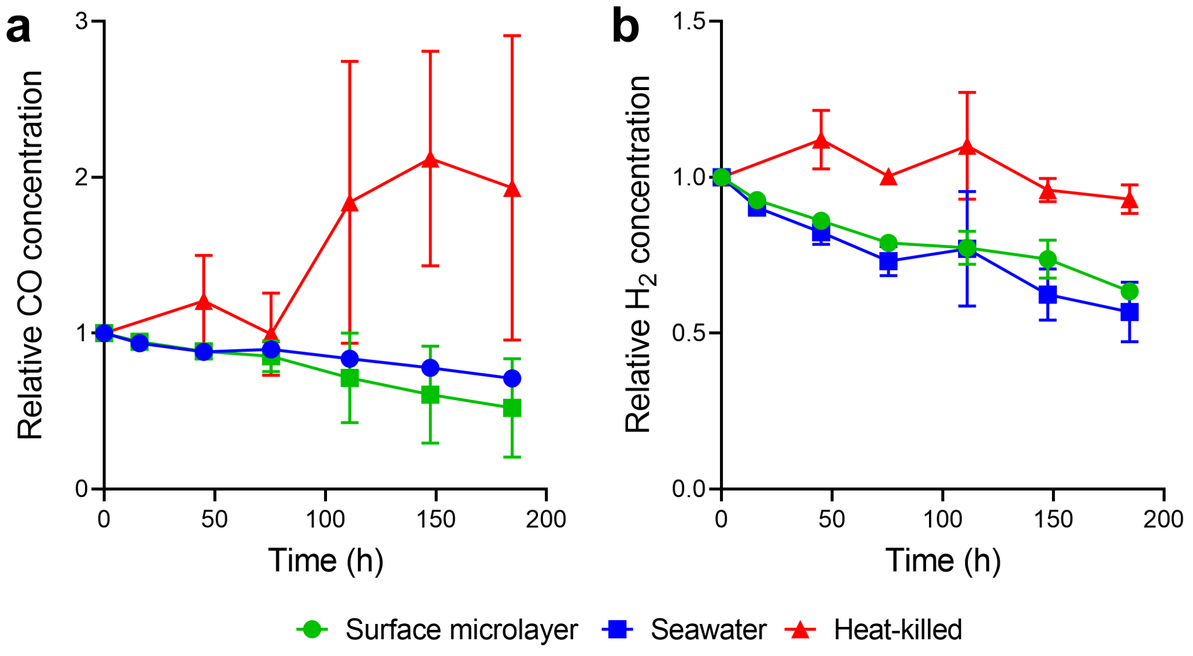

Figure S4. Community composition of marine microbial communities based on metagenomes. The top 10 bacterial and archaeal orders with highest mean relative abundance across all samples are shown. Relative abundances are based on the assembly and classification of the 16S rRNA gene by PhyloFlash. PPB = Port Phillip Bay, SML = Surface microlayer, STW = Subtropical waters, SAW = Subantarctic waters.

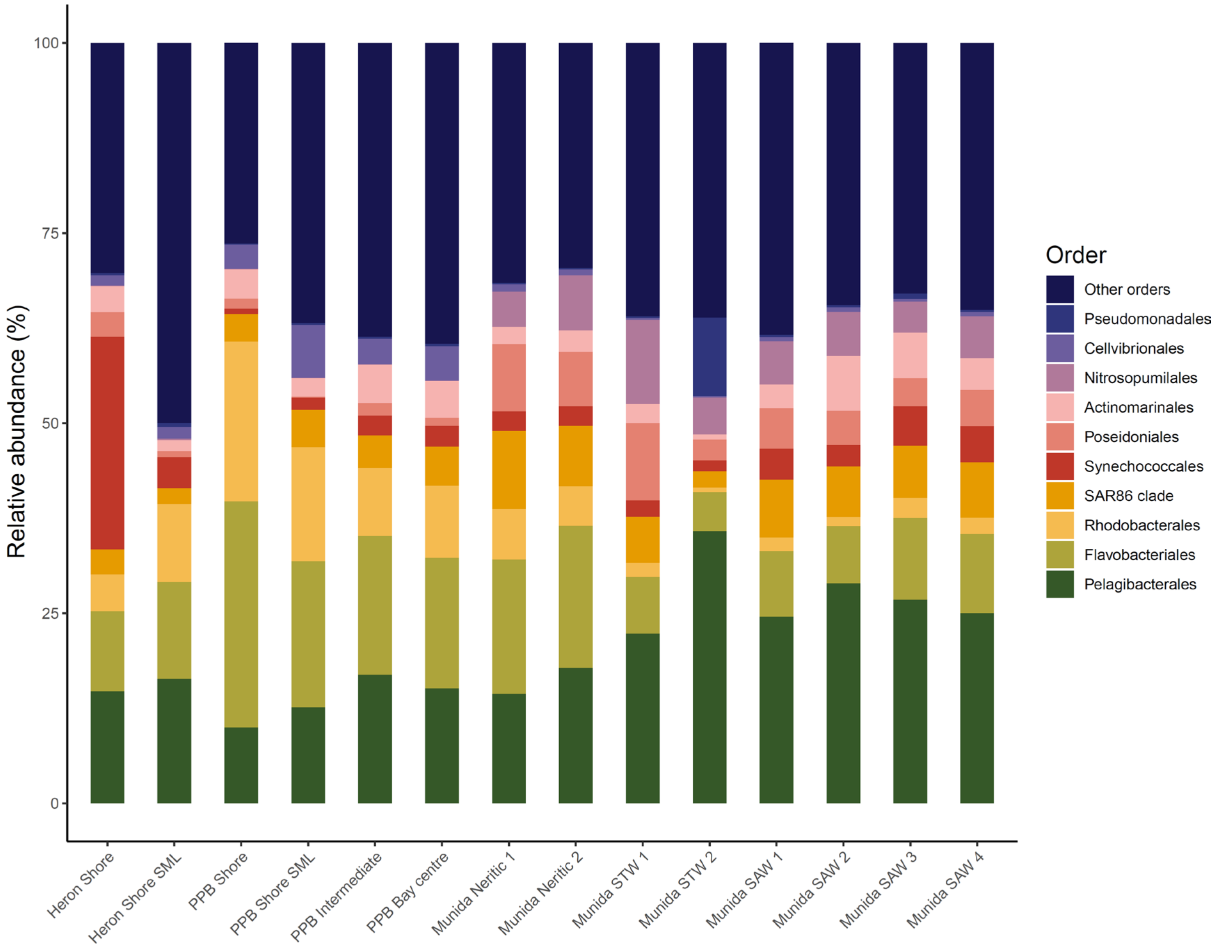

Figure S5. Simple linear correlations between trace gas oxidiser abundance and trace gas oxidation rates. H_2 and CO oxidizer abundance are based on the proportion of bacteria encoding H_2-uptake hydrogenases and form I CO dehydrogenases in the metagenomes. H_2 and CO oxidation rates are calculated based on bulk oxidation rates at measured *in situ* concentrations as per **Table S1**. Pearson's R^2 values show goodness of fit and *p* values confirm each slope significantly deviates from zero.

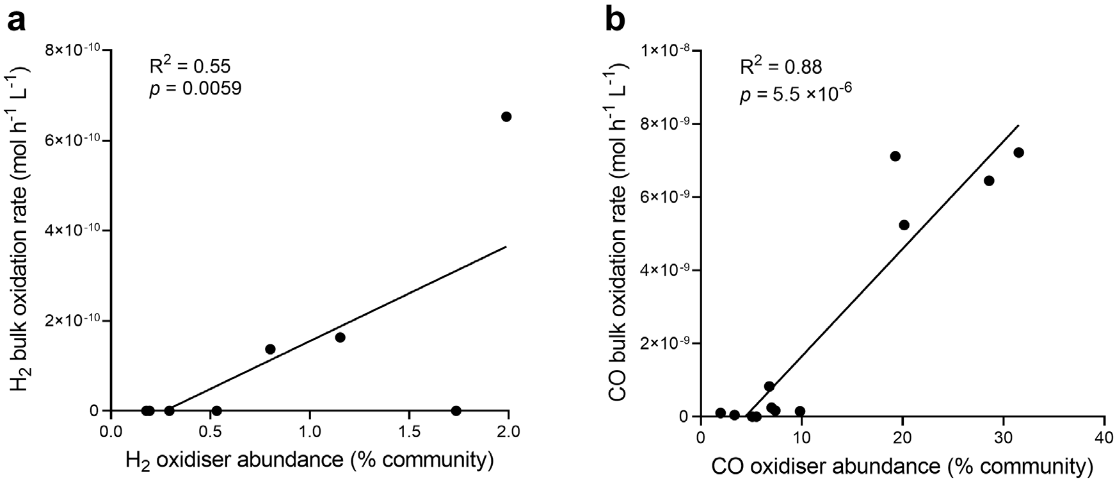

Figure S6. Distribution of metabolic genes in marine bacteria from the three study sites. Bubble plot showing metabolic potential of the 110 metagenome-assembled genomes (MAGs) constructed from the three study sites. MAGs are summarised at phylum level, with the size of the circle corresponding to the number of genomes in that order with a given gene, and the colour reflecting the percentage of genome completeness. Marker genes are omitted that were not detected in any MAG.

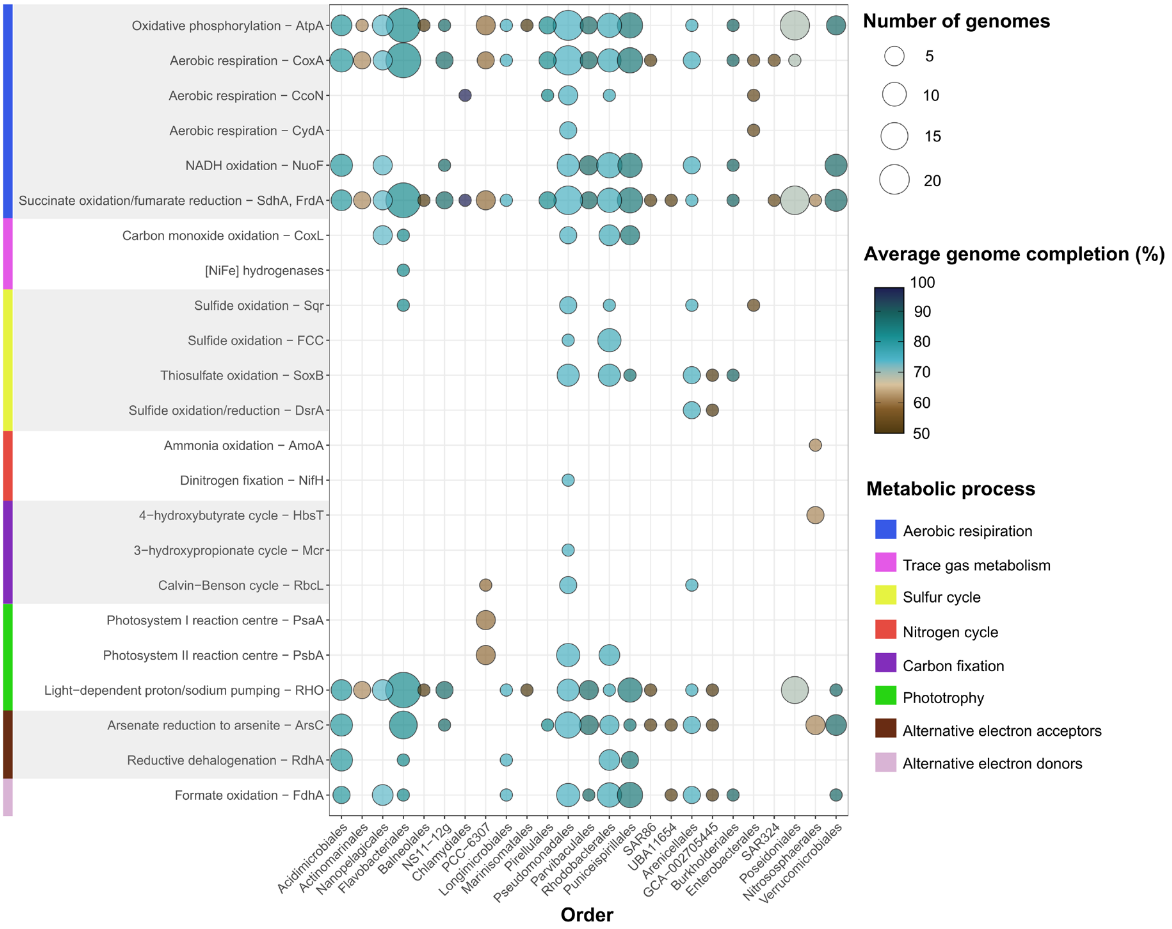

Figure S7. Linear maximum-likelihood phylogenetic tree of marine group 1 and 2 [NiFe]-hydrogenases. Hydrogenase catalytic subunit sequences retrieved from the new MAGs (coloured green) and *Tara* MAGs (coloured green) are shown alongside representative reference sequences (coloured black). Evolutionary history was inferred by using the JTT matrix-based model, the tree was bootstrapped using 50 replicates, and the tree is rooted using the outgroup group 4a [NiFe]-hydrogenase sequence. The tree includes hydrogenase subgroups implicated in aerobic respiration (group 1d, 1f, 1l, 2a), anaerobic respiration (group 1a, 1b, 1c, 1e), and H_2 sensing (group 2b and 2c).

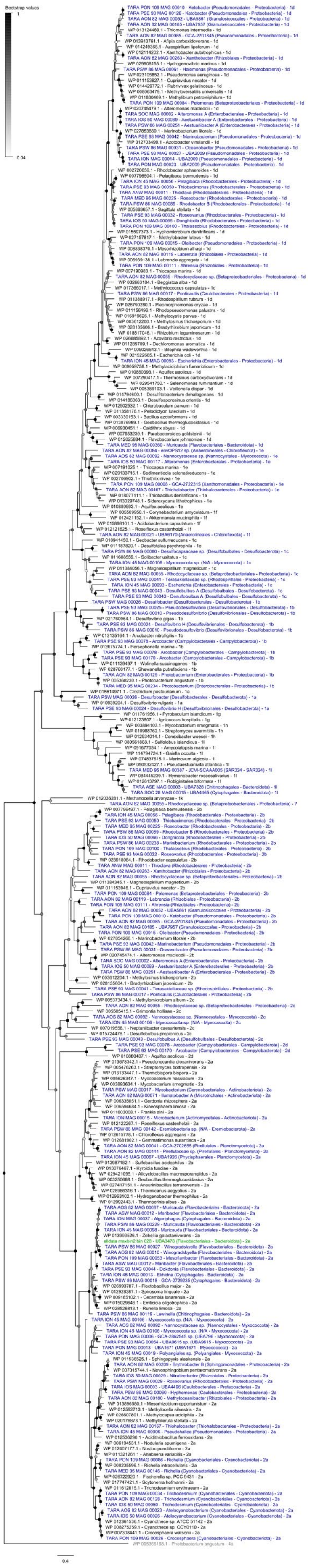

Figure S8. Linear maximum-likelihood phylogenetic tree of marine group 3 and 4 [NiFe]-hydrogenases. Hydrogenase catalytic subunit sequences retrieved from the new MAGs (coloured green) and *Tara* MAGs (coloured green) are shown alongside representative reference sequences (coloured black). Evolutionary history was inferred by using the JTT matrix-based model, the tree was bootstrapped using 50 replicates, and the tree is midpoint-rooted. The tree includes hydrogenase subgroups implicated in fermentation (group 3b, 4a), carbon fixation (group 3d), and energy conversion (group 4e).

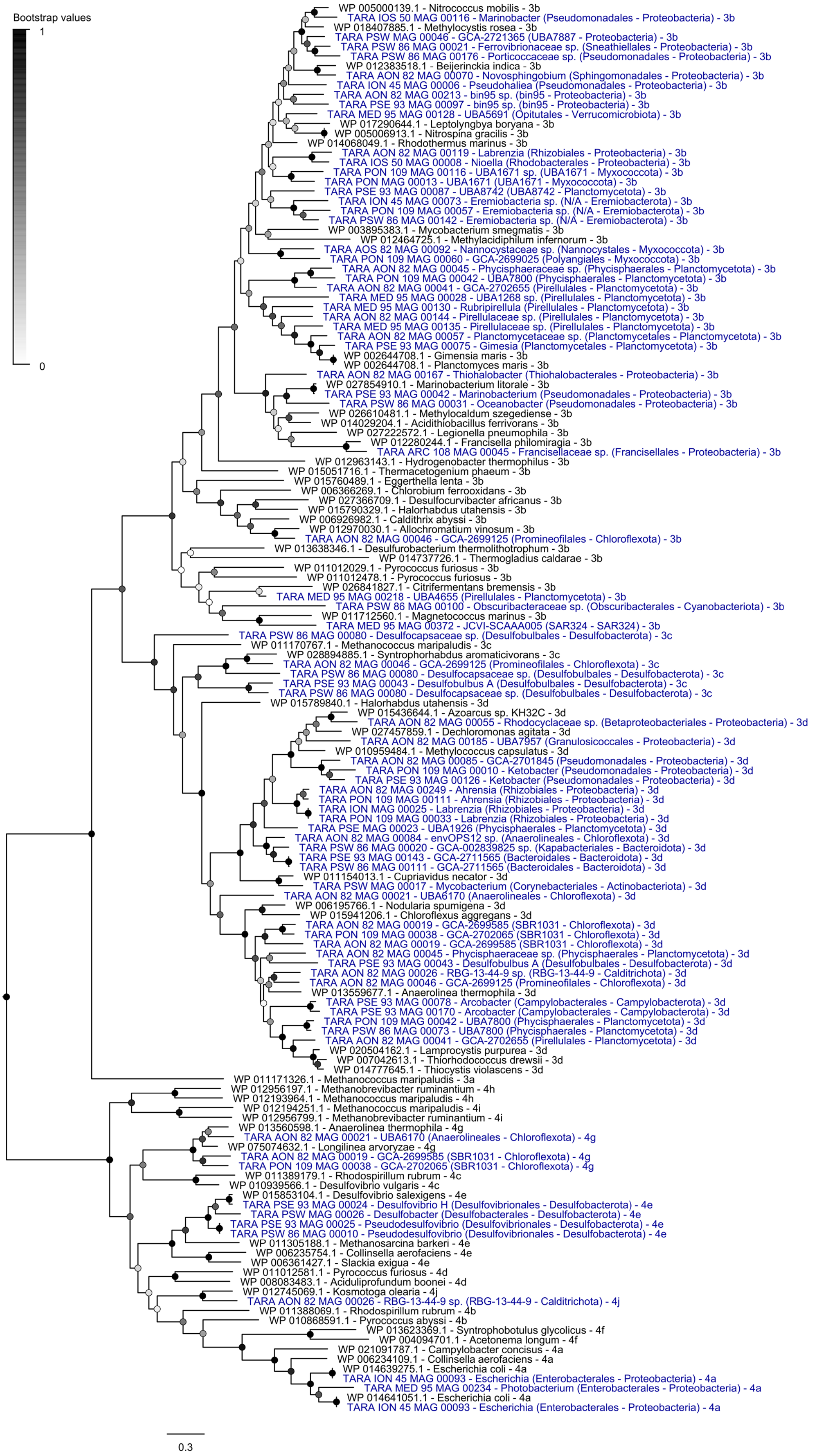

Bootstrap values
1
0
WP 005000139.1 - Nitrococcus mobilis - 3b
TARA IOS 50 MAG 00116 - Marinobacter (Pseudomonadales - Proteobacteria) - 3b
WP 018407885.1 - Methylocystis rosea - 3b
TARA PSW MAG 00046 - GCA-2721365 (UBA7887 - Proteobacteria) - 3b
TARA PSW 86 MAG 00021 - Ferrovibrionaceae sp. (Sneathiellales - Proteobacteria) - 3b
TARA PSW 86 MAG 00176 - Porticoccaceae sp. (Pseudomonadales - Proteobacteria) - 3b
WP 012383518.1 - Beijerinckia indica - 3b
TARA AON 82 MAG 00070 - Novosphingobium (Sphingomonadales - Proteobacteria) - 3b
TARA ION 45 MAG 00006 - Pseudohaliea (Pseudomonadales - Proteobacteria) - 3b
TARA AON 82 MAG 00213 - bin95 sp. (bin95 - Proteobacteria) - 3b
TARA PSE 93 MAG 00097 - bin95 sp. (bin95 - Proteobacteria) - 3b
TARA MED 95 MAG 00128 - UBA5691 (Opitutales - Verrucomicrobiota) - 3b
WP 017290644.1 - Leptolyngbya boryana - 3b
WP 005006913.1 - Nitrospina gracilis - 3b
WP 014068049.1 - Rhodothermus marinus - 3b
TARA AON 82 MAG 00119 - Labrenzia (Rhizobiales - Proteobacteria) - 3b
TARA IOS 50 MAG 00008 - Nioella (Rhodobacterales - Proteobacteria) - 3b
TARA PON 109 MAG 00116 - UBA1671 sp. (UBA1671 - Myxococcota) - 3b
TARA PON MAG 00013 - UBA1671 (UBA1671 - Myxococcota) - 3b
TARA PSE 93 MAG 00087 - UBA8742 (UBA8742 - Planctomycetota) - 3b
TARA ION 45 MAG 00073 - Eremiobacteria sp. (N/A - Eremiobacterota) - 3b
TARA PON 109 MAG 00057 - Eremiobacteria sp. (N/A - Eremiobacterota) - 3b
TARA PSW 86 MAG 00142 - Eremiobacteria sp. (N/A - Eremiobacterota) - 3b
WP 003895383.1 - Mycobacterium smegmatis - 3b
WP 012464725.1 - Methylacidiphilum infernorum - 3b
TARA AOS 82 MAG 00092 - Nannocystaceae sp. (Nannocystales - Myxococcota) - 3b
TARA PON 109 MAG 00060 - GCA-2699025 (Polyangiales - Myxococcota) - 3b
TARA AON 82 MAG 00045 - Phycisphaeraceae sp. (Phycisphaerales - Planctomycetota) - 3b
TARA PON 109 MAG 00042 - UBA7800 (Phycisphaerales - Planctomycetota) - 3b
TARA AON 82 MAG 00041 - GCA-2702655 (Pirellulales - Planctomycetota) - 3b
TARA MED 95 MAG 00028 - UBA1268 sp. (Pirellulales - Planctomycetota) - 3b
TARA MED 95 MAG 00130 - Rubripirellula (Pirellulales - Planctomycetota) - 3b
TARA AON 82 MAG 00144 - Pirellulaceae sp. (Pirellulales - Planctomycetota) - 3b
TARA MED 95 MAG 00135 - Pirellulaceae sp. (Pirellulales - Planctomycetota) - 3b
TARA AON 82 MAG 00057 - Planctomycetaceae sp. (Planctomycetales - Planctomycetota) - 3b
TARA PSE 93 MAG 00075 - Gimesia (Planctomycetales - Planctomycetota) - 3b
WP 002644708.1 - Gimensia maris - 3b
WP 002644708.1 - Planctomyces maris - 3b
TARA AON 82 MAG 00167 - Thiohalobacter (Thiohalobacterales - Proteobacteria) - 3b
WP 027854910.1 - Marinobacterium litorale - 3b
TARA PSE 93 MAG 00042 - Marinobacterium (Pseudomonadales - Proteobacteria) - 3b
TARA PSW 86 MAG 00031 - Oceanobacter (Pseudomonadales - Proteobacteria) - 3b
WP 026610481.1 - Methylocaldum szegediense - 3b
WP 014029204.1 - Acidithiobacillus ferrivorans - 3b
WP 027222572.1 - Legionella pneumophila - 3b
WP 012280244.1 - Francisella philomiragia - 3b
TARA ARC 108 MAG 00045 - Francisellaceae sp. (Francisellales - Proteobacteria) - 3b
WP 012963143.1 - Hydrogenobacter thermophilus - 3b
WP 015051716.1 - Thermacetogenium phaeum - 3b
WP 015760489.1 - Eggerthella lenta - 3b
WP 006366269.1 - Chlorobium ferrooxidans - 3b
WP 027366709.1 - Desulfocurvibacter africanus - 3b
WP 015790329.1 - Halorhabdus utahensis - 3b
WP 006926982.1 - Caldithrix abyssi - 3b
WP 012970030.1 - Allochromatium vinosum - 3b
TARA AON 82 MAG 00046 - GCA-2699125 (Promineofilales - Chloroflexota) - 3b
WP 013638346.1 - Desulfurobacterium thermolithotrophum - 3b
WP 014737726.1 - Thermogladius caldarae - 3b
WP 011012029.1 - Pyrococcus furiosus - 3b
WP 011012478.1 - Pyrococcus furiosus - 3b
WP 026841827.1 - Citrifermentans bremensis - 3b
TARA MED 95 MAG 00218 - UBA4655 (Pirellulales - Planctomycetota) - 3b
TARA PSW 86 MAG 00100 - Obscuribacteraceae sp. (Obscuribacterales - Cyanobacteriota) - 3b
WP 011712560.1 - Magnetococcus marinus - 3b
TARA MED 95 MAG 00372 - JCVI-SCAAA005 (SAR324 - SAR324) - 3b
TARA PSW 86 MAG 00080 - Desulfocapsaceae sp. (Desulfobulbales - Desulfobacterota) - 3c
WP 011170767.1 - Methanococcus maripaludis - 3c
WP 028894885.1 - Syntrophorhabdus aromaticivorans - 3c
TARA AON 82 MAG 00046 - GCA-2699125 (Promineofilales - Chloroflexota) - 3c
TARA PSW 86 MAG 00080 - Desulfocapsaceae sp. (Desulfobulbales - Desulfobacterota) - 3c
TARA PSE 93 MAG 00043 - Desulfobulbus A (Desulfobulbales - Desulfobacterota) - 3c
TARA PSW 86 MAG 00080 - Desulfocapsaceae sp. (Desulfobulbales - Desulfobacterota) - 3c
WP 015789840.1 - Halorhabdus utahensis - 3d
WP 015436644.1 - Azoarcus sp. KH32C - 3d
TARA AON 82 MAG 00055 - Rhodocyclaceae sp. (Betaproteobacteriales - Proteobacteria) - 3d
WP 027457859.1 - Dechloromonas agitata - 3d
TARA AON 82 MAG 00185 - UBA7957 (Granulosicoccales - Proteobacteria) - 3d
WP 010959484.1 - Methylococcus capsulatus - 3d
TARA AON 82 MAG 00085 - GCA-2701845 (Pseudomonadales - Proteobacteria) - 3d
TARA PON 109 MAG 00010 - Ketobacter (Pseudomonadales - Proteobacteria) - 3d
TARA PSE 93 MAG 00126 - Ketobacter (Pseudomonadales - Proteobacteria) - 3d
TARA AON 82 MAG 00249 - Ahrensia (Rhizobiales - Proteobacteria) - 3d
TARA PON 109 MAG 00111 - Ahrensia (Rhizobiales - Proteobacteria) - 3d
TARA ION MAG 00025 - Labrenzia (Rhizobiales - Proteobacteria) - 3d
TARA PON 109 MAG 00033 - Labrenzia (Rhizobiales - Proteobacteria) - 3d
TARA PSE MAG 00023 - UBA1926 (Phycisphaerales - Planctomycetota) - 3d
TARA AON 82 MAG 00084 - envOPS12 sp. (Anaerolineales - Chloroflexota) - 3d
TARA PSW 86 MAG 00020 - GCA-002839825 sp. (Kapabacteriales - Bacteroidota) - 3d
TARA PSE 93 MAG 00143 - GCA-2711565 (Bacteroidales - Bacteroidota) - 3d
TARA PSW 86 MAG 00111 - GCA-2711565 (Bacteroidales - Bacteroidota) - 3d
WP 011154013.1 - Cupriavidus necator - 3d
TARA PSW MAG 00017 - Mycobacterium (Corynebacteriales - Actinobacteriota) - 3d
TARA AON 82 MAG 00021 - UBA6170 (Anaerolineales - Chloroflexota) - 3d
WP 006195766.1 - Nodularia spumigena - 3d
WP 015941206.1 - Chloroflexus aggregans - 3d
TARA AON 82 MAG 00019 - GCA-2699585 (SBR1031 - Chloroflexota) - 3d
TARA PON 109 MAG 00038 - GCA-2702065 (SBR1031 - Chloroflexota) - 3d
TARA AON 82 MAG 00019 - GCA-2699585 (SBR1031 - Chloroflexota) - 3d
TARA AON 82 MAG 00045 - Phycisphaeraceae sp. (Phycisphaerales - Planctomycetota) - 3d
TARA PSE 93 MAG 00043 - Desulfobulbus A (Desulfobulbales - Desulfobacterota) - 3d
TARA AON 82 MAG 00026 - RBG-13-44-9 sp. (RBG-13-44-9 - Calditrichota) - 3d
TARA AON 82 MAG 00046 - GCA-2699125 (Promineofilales - Chloroflexota) - 3d
WP 013559677.1 - Anaerolinea thermophila - 3d
TARA PSE 93 MAG 00078 - Arcobacter (Campylobacterales - Campylobacterota) - 3d
TARA PSE 93 MAG 00170 - Arcobacter (Campylobacterales - Campylobacterota) - 3d
TARA PON 109 MAG 00042 - UBA7800 (Phycisphaerales - Planctomycetota) - 3d
TARA PSW 86 MAG 00073 - UBA7800 (Phycisphaerales - Planctomycetota) - 3d
TARA AON 82 MAG 00041 - GCA-2702655 (Pirellulales - Planctomycetota) - 3d
WP 020504162.1 - Lamprocystis purpurea - 3d
WP 007042613.1 - Thiorhodococcus drewsii - 3d
WP 014777645.1 - Thiocystis violascens - 3d
WP 011171326.1 - Methanococcus maripaludis - 3a
WP 012956197.1 - Methanobrevibacter ruminantium - 4h
WP 012193964.1 - Methanococcus maripaludis - 4h
WP 012194251.1 - Methanococcus maripaludis - 4i
WP 012956799.1 - Methanobrevibacter ruminantium - 4i
WP 013560598.1 - Anaerolinea thermophila - 4g
TARA AON 82 MAG 00021 - UBA6170 (Anaerolineales - Chloroflexota) - 4g
WP 075074632.1 - Longilinea arvoryzae - 4g
TARA AON 82 MAG 00019 - GCA-2699585 (SBR1031 - Chloroflexota) - 4g
TARA PON 109 MAG 00038 - GCA-2702065 (SBR1031 - Chloroflexota) - 4g
WP 011389179.1 - Rhodospirillum rubrum - 4c
WP 010939566.1 - Desulfovibrio vulgaris - 4c
WP 015853104.1 - Desulfovibrio salexigens - 4e
TARA PSE 93 MAG 00024 - Desulfovibrio H (Desulfovibrionales - Desulfobacterota) - 4e
TARA PSW MAG 00026 - Desulfobacter (Desulfobacterales - Desulfobacterota) - 4e
TARA PSE 93 MAG 00025 - Pseudodesulfovibrio (Desulfovibrionales - Desulfobacterota) - 4e
TARA PSW 86 MAG 00010 - Pseudodesulfovibrio (Desulfovibrionales - Desulfobacterota) - 4e
WP 011305188.1 - Methanosarcina barkeri - 4e
WP 006235754.1 - Collinsella aerofaciens - 4e
WP 006361427.1 - Slackia exigua - 4e
WP 011012581.1 - Pyrococcus furiosus - 4d
WP 008083483.1 - Aciduliprofundum boonei - 4d
WP 012745069.1 - Kosmotoga olearia - 4j
TARA AON 82 MAG 00026 - RBG-13-44-9 sp. (RBG-13-44-9 - Calditrichota) - 4j
WP 011388069.1 - Rhodospirillum rubrum - 4b
WP 010868591.1 - Pyrococcus abyssi - 4b
WP 013623369.1 - Syntrophobotulus glycolicus - 4f
WP 004094701.1 - Acetonema longum - 4f
WP 021091787.1 - Campylobacter concisus - 4a
WP 006234109.1 - Collinsella aerofaciens - 4a
WP 014639275.1 - Escherichia coli - 4a
TARA ION 45 MAG 00093 - Escherichia (Enterobacterales - Proteobacteria) - 4a
TARA MED 95 MAG 00234 - Photobacterium (Enterobacterales - Proteobacteria) - 4a
WP 014641051.1 - Escherichia coli - 4a
TARA ION 45 MAG 00093 - Escherichia (Enterobacterales - Proteobacteria) - 4a
0.3

Figure S9. Linear maximum-likelihood phylogenetic tree of marine carbon monoxide dehydrogenases. Catalytic subunit sequences retrieved from the new MAGs (coloured green) and *Tara* MAGs (coloured green) are shown alongside representative reference sequences (coloured black). Evolutionary history was inferred by using the JTT matrix-based model, the tree was bootstrapped using 50 replicates, and the tree is rooted using outgroups including the form II CO dehydrogenase.

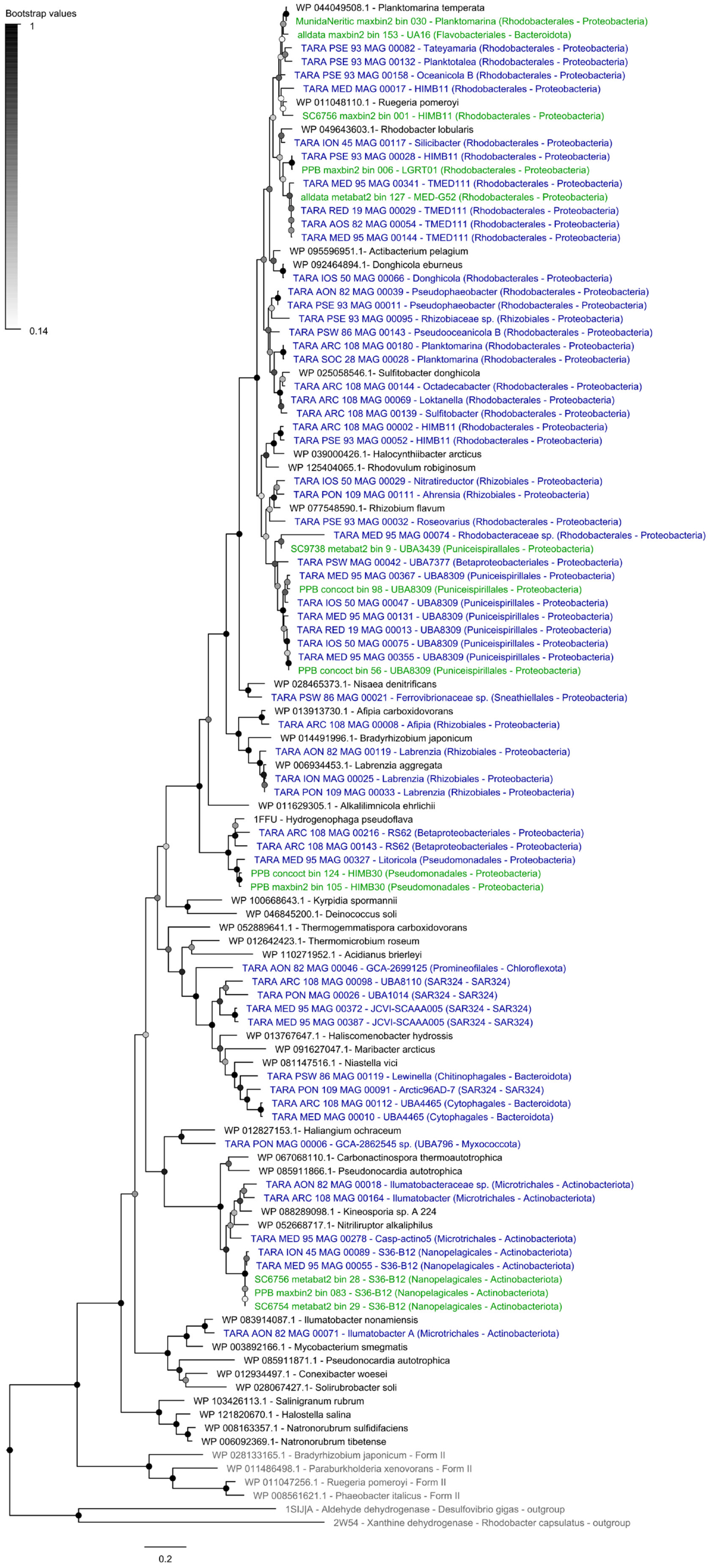

Bootstrap values
1
0.14
WP 044049508.1 - Planktomarina temperata
MunidaNeritic maxbin2 bin 030 - Planktomarina (Rhodobacterales - Proteobacteria)
alldata maxbin2 bin 153 - UA16 (Flavobacteriales - Bacteroidota)
TARA PSE 93 MAG 00082 - Tateyamaria (Rhodobacterales - Proteobacteria)
TARA PSE 93 MAG 00132 - Planktotalea (Rhodobacterales - Proteobacteria)
TARA PSE 93 MAG 00158 - Oceanicola B (Rhodobacterales - Proteobacteria)
TARA MED MAG 00017 - HIMB11 (Rhodobacterales - Proteobacteria)
WP 011048110.1 - Ruegeria pomeroyi
SC6756 maxbin2 bin 001 - HIMB11 (Rhodobacterales - Proteobacteria)
WP 049643603.1- Rhodobacter lobularis
TARA ION 45 MAG 00117 - Silicibacter (Rhodobacterales - Proteobacteria)
TARA PSE 93 MAG 00028 - HIMB11 (Rhodobacterales - Proteobacteria)
PPB maxbin2 bin 006 - LGRT01 (Rhodobacterales - Proteobacteria)
TARA MED 95 MAG 00341 - TMED111 (Rhodobacterales - Proteobacteria)
alldata metabat2 bin 127 - MED-G52 (Rhodobacterales - Proteobacteria)
TARA RED 19 MAG 00029 - TMED111 (Rhodobacterales - Proteobacteria)
TARA AOS 82 MAG 00054 - TMED111 (Rhodobacterales - Proteobacteria)
TARA MED 95 MAG 00144 - TMED111 (Rhodobacterales - Proteobacteria)
WP 095596951.1- Actibacterium pelagium
WP 092464894.1- Donghicola eburneus
TARA IOS 50 MAG 00066 - Donghicola (Rhodobacterales - Proteobacteria)
TARA AON 82 MAG 00039 - Pseudophaeobacter (Rhodobacterales - Proteobacteria)
TARA PSE 93 MAG 00011 - Pseudophaeobacter (Rhodobacterales - Proteobacteria)
TARA PSE 93 MAG 00095 - Rhizobiaceae sp. (Rhizobiales - Proteobacteria)
TARA PSW 86 MAG 00143 - Pseudooceanicola B (Rhodobacterales - Proteobacteria)
TARA ARC 108 MAG 00180 - Planktomarina (Rhodobacterales - Proteobacteria)
TARA SOC 28 MAG 00028 - Planktomarina (Rhodobacterales - Proteobacteria)
WP 025058546.1- Sulfitobacter donghicola
TARA ARC 108 MAG 00144 - Octadecabacter (Rhodobacterales - Proteobacteria)
TARA ARC 108 MAG 00069 - Loktanella (Rhodobacterales - Proteobacteria)
TARA ARC 108 MAG 00139 - Sulfitobacter (Rhodobacterales - Proteobacteria)
TARA ARC 108 MAG 00002 - HIMB11 (Rhodobacterales - Proteobacteria)
TARA PSE 93 MAG 00052 - HIMB11 (Rhodobacterales - Proteobacteria)
WP 039000426.1- Halocynthiibacter arcticus
WP 125404065.1- Rhodovulum robiginosum
TARA IOS 50 MAG 00029 - Nitratireductor (Rhizobiales - Proteobacteria)
TARA PON 109 MAG 00111 - Ahrensia (Rhizobiales - Proteobacteria)
WP 077548590.1- Rhizobium flavum
TARA PSE 93 MAG 00032 - Roseovarius (Rhodobacterales - Proteobacteria)
TARA MED 95 MAG 00074 - Rhodobacteraceae sp. (Rhodobacterales - Proteobacteria)
SC9738 metabat2 bin 9 - UBA3439 (Puniceispirillales - Proteobacteria)
TARA PSW MAG 00042 - UBA7377 (Betaproteobacteriales - Proteobacteria)
TARA MED 95 MAG 00367 - UBA8309 (Puniceispirillales - Proteobacteria)
PPB concoct bin 98 - UBA8309 (Puniceispirillales - Proteobacteria)
TARA IOS 50 MAG 00047 - UBA8309 (Puniceispirillales - Proteobacteria)
TARA MED 95 MAG 00131 - UBA8309 (Puniceispirillales - Proteobacteria)
TARA RED 19 MAG 00013 - UBA8309 (Puniceispirillales - Proteobacteria)
TARA IOS 50 MAG 00075 - UBA8309 (Puniceispirillales - Proteobacteria)
TARA MED 95 MAG 00355 - UBA8309 (Puniceispirillales - Proteobacteria)
PPB concoct bin 56 - UBA8309 (Puniceispirillales - Proteobacteria)
WP 028465373.1- Nisaea denitrificans
TARA PSW 86 MAG 00021 - Ferrovibrionaceae sp. (Sneathiellales - Proteobacteria)
WP 013913730.1 - Afipia carboxidovorans
TARA ARC 108 MAG 00008 - Afipia (Rhizobiales - Proteobacteria)
WP 014491996.1- Bradyrhizobium japonicum
TARA AON 82 MAG 00119 - Labrenzia (Rhizobiales - Proteobacteria)
WP 006934453.1- Labrenzia aggregata
TARA ION MAG 00025 - Labrenzia (Rhizobiales - Proteobacteria)
TARA PON 109 MAG 00033 - Labrenzia (Rhizobiales - Proteobacteria)
WP 011629305.1 - Alkalilimnicola ehrlichii
1FFU - Hydrogenophaga pseudoflava
TARA ARC 108 MAG 00216 - RS62 (Betaproteobacteriales - Proteobacteria)
TARA ARC 108 MAG 00143 - RS62 (Betaproteobacteriales - Proteobacteria)
TARA MED 95 MAG 00327 - Litoricola (Pseudomonadales - Proteobacteria)
PPB concoct bin 124 - HIMB30 (Pseudomonadales - Proteobacteria)
PPB maxbin2 bin 105 - HIMB30 (Pseudomonadales - Proteobacteria)
WP 100668643.1 - Kyrpidia spormannii
WP 046845200.1- Deinococcus soli
WP 052889641.1 - Thermogemmatispora carboxidovorans
WP 012642423.1- Thermomicrobium roseum
WP 110271952.1 - Acidianus brierleyi
TARA AON 82 MAG 00046 - GCA-2699125 (Promineofilales - Chloroflexota)
TARA ARC 108 MAG 00098 - UBA8110 (SAR324 - SAR324)
TARA PON MAG 00026 - UBA1014 (SAR324 - SAR324)
TARA MED 95 MAG 00372 - JCVI-SCAAA005 (SAR324 - SAR324)
TARA MED 95 MAG 00387 - JCVI-SCAAA005 (SAR324 - SAR324)
WP 013767647.1 - Haliscomenobacter hydrossis
WP 091627047.1- Maribacter arcticus
WP 081147516.1 - Niastella vici
TARA PSW 86 MAG 00119 - Lewinella (Chitinophagales - Bacteroidota)
TARA PON 109 MAG 00091 - Arctic96AD-7 (SAR324 - SAR324)
TARA ARC 108 MAG 00112 - UBA4465 (Cytophagales - Bacteroidota)
TARA MED MAG 00010 - UBA4465 (Cytophagales - Bacteroidota)
WP 012827153.1- Haliangium ochraceum
TARA PON MAG 00006 - GCA-2862545 sp. (UBA796 - Myxococcota)
WP 067068110.1- Carbonactinospora thermoautotrophica
WP 085911866.1- Pseudonocardia autotrophica
TARA AON 82 MAG 00018 - Ilumatobacteraceae sp. (Microtrichales - Actinobacteriota)
TARA ARC 108 MAG 00164 - Ilumatobacter (Microtrichales - Actinobacteriota)
WP 088289098.1 - Kineosporia sp. A 224
WP 052668717.1- Nitriliruptor alkaliphilus
TARA MED 95 MAG 00278 - Casp-actino5 (Microtrichales - Actinobacteriota)
TARA ION 45 MAG 00089 - S36-B12 (Nanopelagicales - Actinobacteriota)
TARA MED 95 MAG 00055 - S36-B12 (Nanopelagicales - Actinobacteriota)
SC6756 metabat2 bin 28 - S36-B12 (Nanopelagicales - Actinobacteriota)
PPB maxbin2 bin 083 - S36-B12 (Nanopelagicales - Actinobacteriota)
SC6754 metabat2 bin 29 - S36-B12 (Nanopelagicales - Actinobacteriota)
WP 083914087.1 - Ilumatobacter nonamiensis
TARA AON 82 MAG 00071 - Ilumatobacter A (Microtrichales - Actinobacteriota)
WP 003892166.1 - Mycobacterium smegmatis
WP 085911871.1 - Pseudonocardia autotrophica
WP 012934497.1 - Conexibacter woesei
WP 028067427.1- Solirubrobacter soli
WP 103426113.1 - Salinigranum rubrum
WP 121820670.1 - Halostella salina
WP 008163357.1 - Natronorubrum sulfidifaciens
WP 006092369.1- Natronorubrum tibetense
WP 028133165.1 - Bradyrhizobium japonicum - Form II
WP 011486498.1 - Paraburkholderia xenovorans - Form II
WP 011047256.1 - Ruegeria pomeroyi - Form II
WP 008561621.1 - Phaeobacter italicus - Form II
1SIJ|A - Aldehyde dehydrogenase - Desulfovibrio gigas - outgroup
2W54 - Xanthine dehydrogenase - Rhodobacter capsulatus - outgroup
0.2

Figure S10. Correlation matrix of environmental variables based on *Tara* Oceans metadata. Pearson correlations were performed on the original *Tara* Oceans metadata without imputation of missing values: each pairwise correlation was calculated using all complete pairs of observations for that variable.

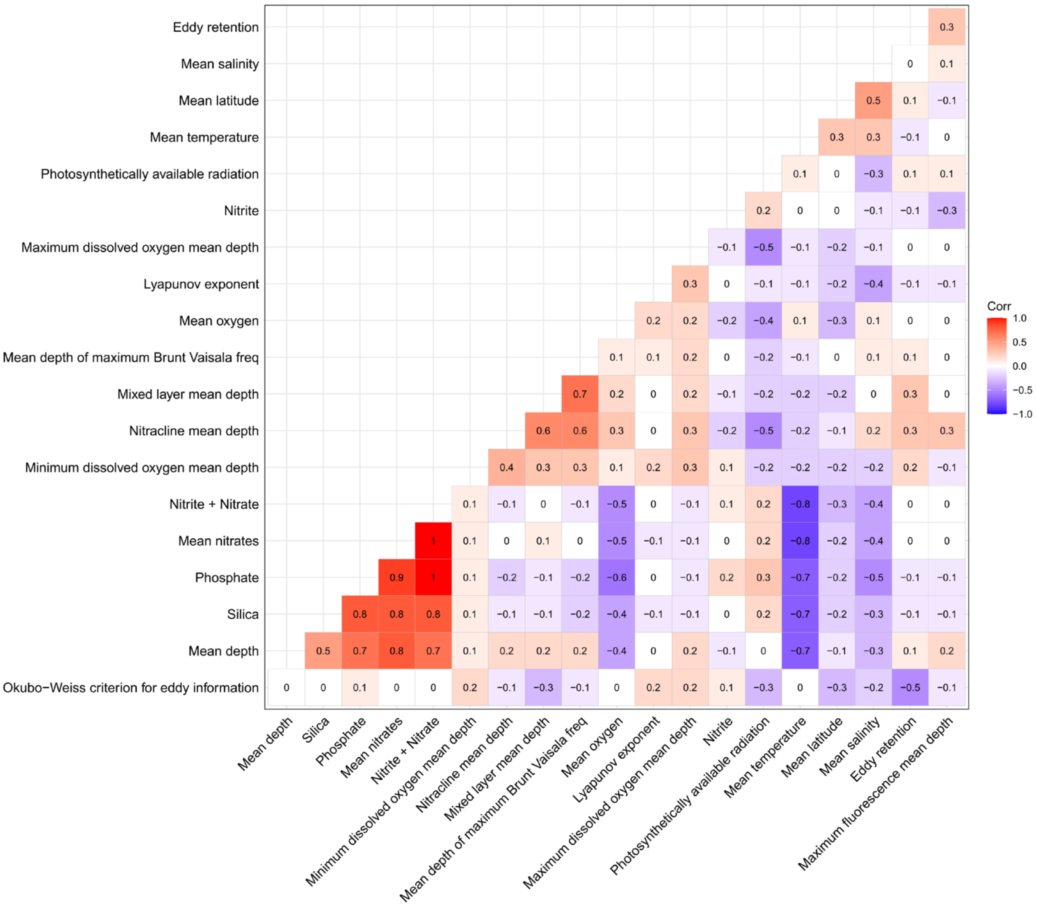

Figure S11. Random forest analysis of strongest environmental predictors of metabolic gene abundance. Results are shown based on the gene copies per organism of energy-converting rhodopsins, CO dehydrogenases, and various [NiFe]-hydrogenases based on the *Tara* Oceans metagenomes and accompanying metadata.

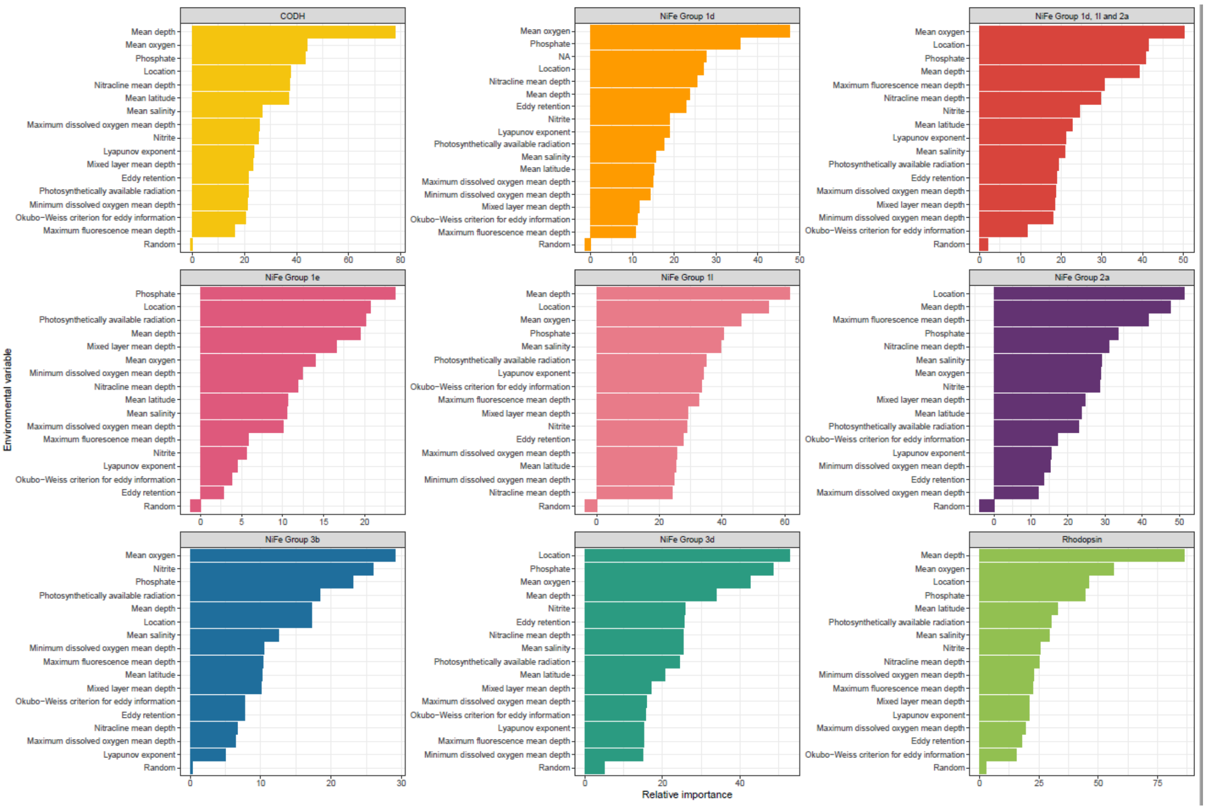

Figure S12. Random forest analysis of strongest environmental predictors of metabolic gene expression. Results are shown based on the reads per kilobase million (RPKM; log-transformed) of energy-converting rhodopsins, CO dehydrogenases, and various [NiFe]-hydrogenases based on the *Tara* Oceans metatranscriptomes and accompanying metadata.

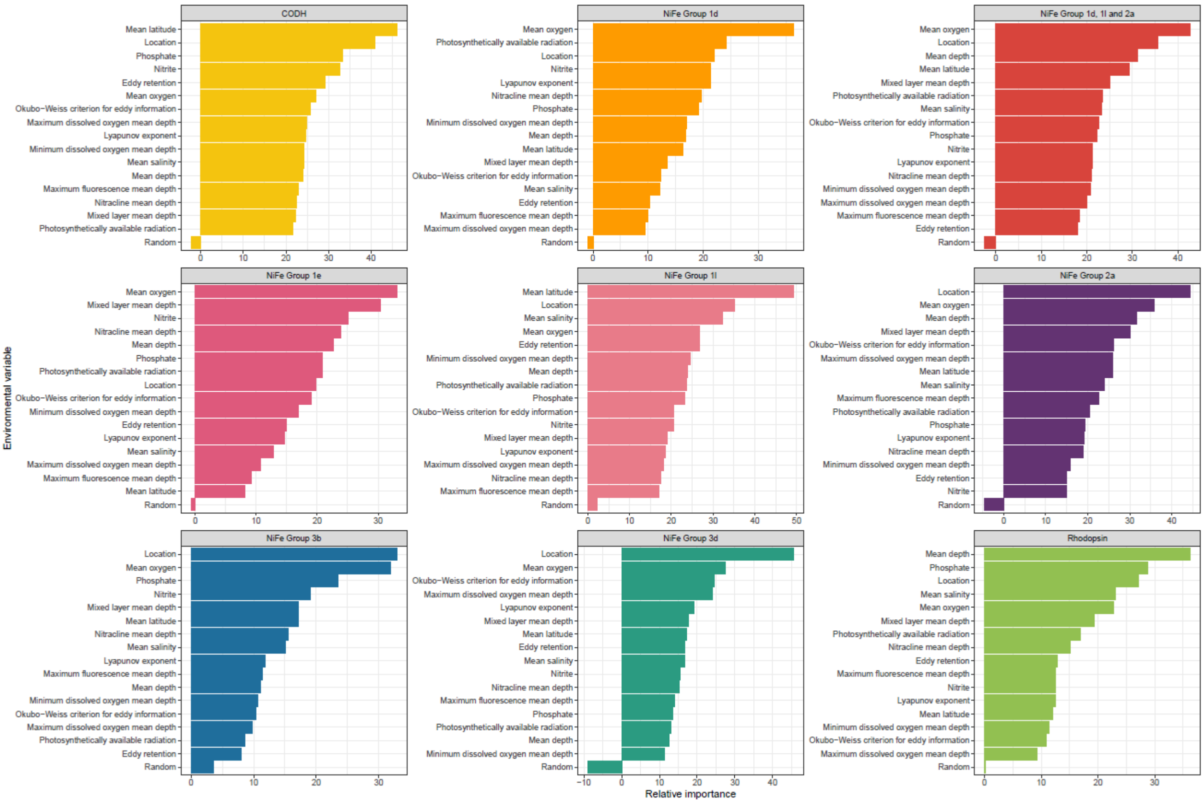

Figure S13. Relationship between latitude and trace gas oxidation capacity in the *Tara* Oceans dataset. Samples are coloured by surface (blue), deep chlorophyll maximum (red) or mesopelagic (green) depth. Gene abundance is represented on the top row, and gene expression on the bottom row.

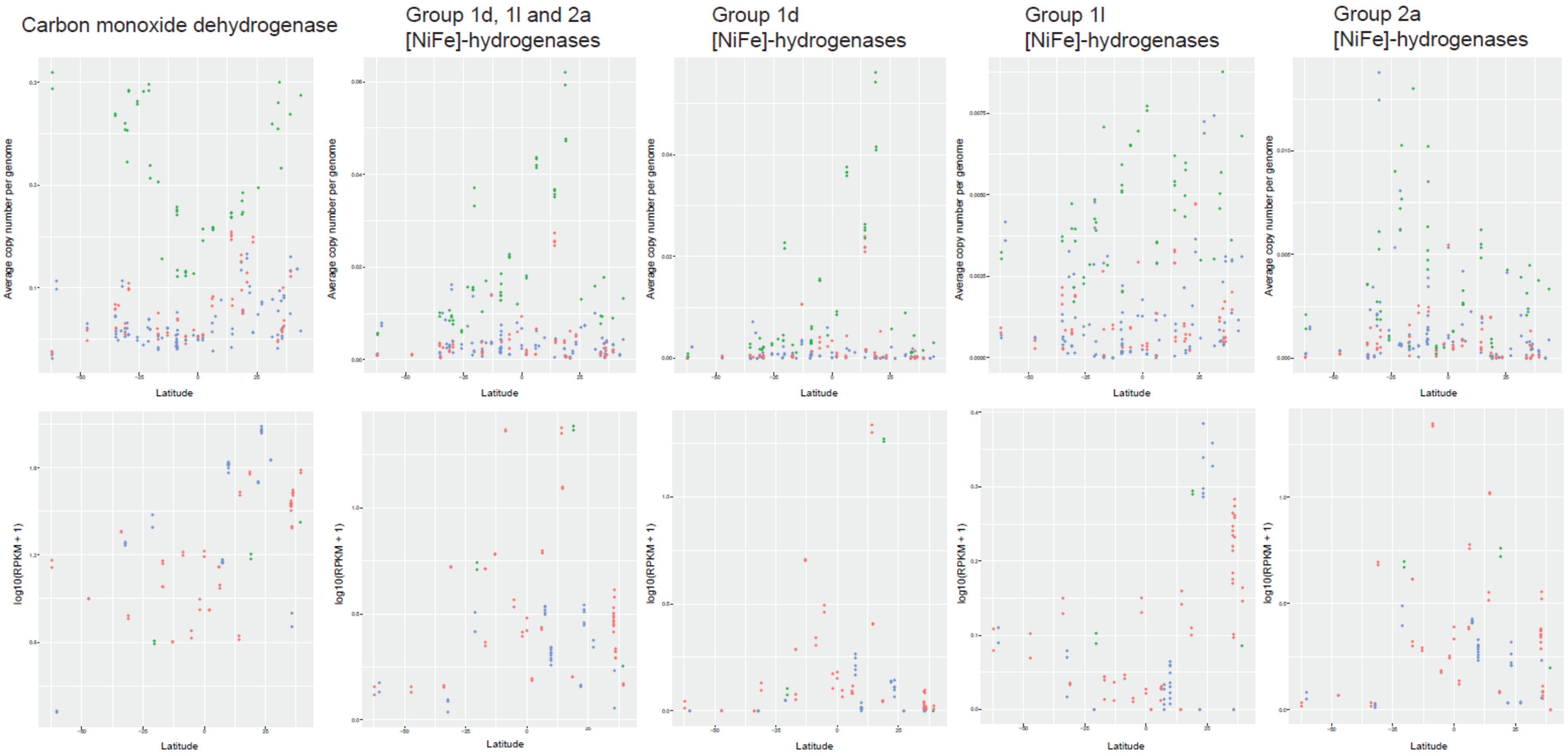

Figure S14. Relationship between oxygen concentration and trace gas oxidation capacity in the *Tara* Oceans dataset. Samples are coloured by surface (blue), deep chlorophyll maximum (red) or mesopelagic (green) depth. Gene abundance is represented in the top row, and gene expression on the bottom row.

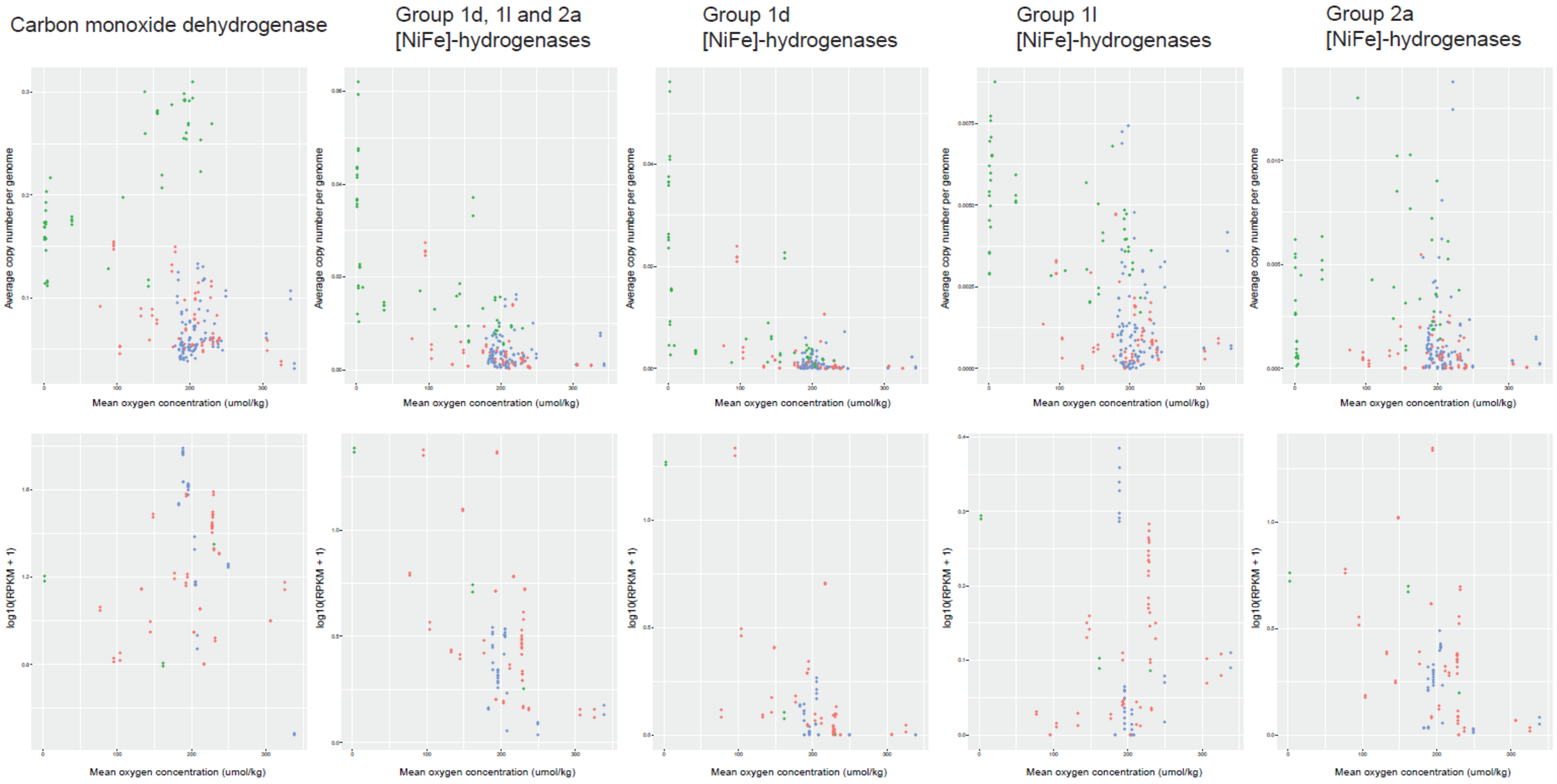

Supplement: Supplementary file 1 — Supplementary Figs. 1–14 and legends for Supplementary Tables 1–6. [file 41564_2023_1322_MOESM1_ESM.pdf]
