# Supplementary material for: Molecular hydrogen in seawater supports growth of diverse marine bacteria
Source: Nat Microbiol. 2023 Feb 6;8(4):581–95. doi: 10.1038/s41564-023-01322-0 (PMC10305171; doi:10.1038/s41564-023-01322-0)
Supplement: Supplementary file 2 — Reporting Summary [file 41564_2023_1322_MOESM2_ESM.pdf]

nature portfolio

Corresponding author(s): Chris Greening
Last updated by author(s): 16/12/22

Reporting Summary

Nature Portfolio wishes to improve the reproducibility of the work that we publish. This form provides structure for consistency and transparency in reporting. For further information on Nature Portfolio policies, see our Editorial Policies and the Editorial Policy Checklist.

Statistics

For all statistical analyses, confirm that the following items are present in the figure legend, table legend, main text, or Methods section.

n/a	Confirmed	
☐	☒	The exact sample size (*n*) for each experimental group/condition, given as a discrete number and unit of measurement
☐	☒	A statement on whether measurements were taken from distinct samples or whether the same sample was measured repeatedly
☐	☒	The statistical test(s) used AND whether they are one- or two-sided<br>*Only common tests should be described solely by name; describe more complex techniques in the Methods section.*
☒	☐	A description of all covariates tested
☒	☐	A description of any assumptions or corrections, such as tests of normality and adjustment for multiple comparisons
☐	☒	A full description of the statistical parameters including central tendency (e.g. means) or other basic estimates (e.g. regression coefficient) AND variation (e.g. standard deviation) or associated estimates of uncertainty (e.g. confidence intervals)
☐	☒	For null hypothesis testing, the test statistic (e.g. *F*, *t*, *r*) with confidence intervals, effect sizes, degrees of freedom and *P* value noted<br>*Give P values as exact values whenever suitable.*
☒	☐	For Bayesian analysis, information on the choice of priors and Markov chain Monte Carlo settings
☒	☐	For hierarchical and complex designs, identification of the appropriate level for tests and full reporting of outcomes
☐	☒	Estimates of effect sizes (e.g. Cohen's *d*, Pearson's *r*), indicating how they were calculated

Our web collection on statistics for biologists contains articles on many of the points above.

Software and code

Policy information about availability of computer code

Data collection	Metagenomics analysis scripts are publicly available at https://github.com/greeninglab/MarineOxidationManuscript.
Data analysis	Databases used to analyse data or against which data was compared to include:<br>- UniProtKB (https://www.uniprot.org/help/uniprotkb)<br>- HydDB (https://services.birc.au.dk/hyddb/)<br>- Curated metabolic marker databases (https://bridges.monash.edu/collections/_/5230745)<br>- GTDB (release 202; https://gtdb.ecogenomic.org/)<br><br>Data were analysed using the following published software:<br>- BBTools suite v38.90<br>- BBMap v38.90<br>- PhyloFlash v3.4<br>- MEGAHIT v1.2.9<br>- MetaBAT2 v2.15.5<br>- MaxBin 2 v2.2.7<br>- CONCOCT v.1.1.0<br>- DAS_Tool v1.1.3<br>- RefineM v0.1.2<br>- CheckM v1.1.3<br>- GTDB-Tk v1.6.0<br>- Prodigal v2.6.3

- CoverM v0.6.1
- DIAMOND v2.0.9
- SingleM v0.13.2
- MEGA 11 v11.0.13 (including the following parameters: ClustalW (v2.0), Jones-Taylor-Thornton (JTT) model, Neighbour-Join statistical algorithm and BioNJ heuristic inference)
- Fastqc v0.11.7
- R v4.2.0 (including the following packages: randomForest, tidyr, dplyr, purrr, ggplot2, MetBrewer and ggcorrplot)
- GraphPad Prism 9
- dRep v3.2.2
- iTOL v6.6

Code availability:
Bash scripts used to process metagenomes, metatranscriptomes and MAGs are publicly available here: https://github.com/GreeningLab/MarineOxidationManuscript.

For manuscripts utilizing custom algorithms or software that are central to the research but not yet described in published literature, software must be made available to editors and reviewers. We strongly encourage code deposition in a community repository (e.g. GitHub). See the Nature Portfolio guidelines for submitting code & software for further information.

Data

Policy information about availability of data

All manuscripts must include a data availability statement. This statement should provide the following information, where applicable:
- Accession codes, unique identifiers, or web links for publicly available datasets
- A description of any restrictions on data availability
- For clinical datasets or third party data, please ensure that the statement adheres to our policy

All raw metagenomes and metagenome-assembled genomes are deposited to the NCBI Sequence Read Archive under the BioProject accession number PRJNA801081. Raw metagenome and metatranscriptome data from the Tara Oceans global dataset were downloaded from the European Nucleotide Archive (https://www.ebi.ac.uk/ena/browser/home), under the following study numbers: PRJEB1787 (metagenomes) and PRJEB6608 (metatranscriptomes). 1888 bacterial and archaeal MAGs generated by Delmont et al. (2021) were downloaded from https://www.genoscope.cns.fr/tara/. The metabolic marker protein database used in this study, which includes reference hydrogenase and carbon monoxide dehydrogenase sequences can be obtained from https://bridges.monash.edu/collections/_/5230745.

Human research participants

Policy information about studies involving human research participants and Sex and Gender in Research.

Reporting on sex and gender	This information was not collected as there were no human research participants involved in this study.
Population characteristics	This information was not collected as there were no human research participants.
Recruitment	This information was not collected as there were no human research participants.
Ethics oversight	This information was not collected as there were no human research participants.

Note that full information on the approval of the study protocol must also be provided in the manuscript.

Field-specific reporting

Please select the one below that is the best fit for your research. If you are not sure, read the appropriate sections before making your selection.

☐ Life sciences ☐ Behavioural & social sciences ☒ Ecological, evolutionary & environmental sciences

For a reference copy of the document with all sections, see nature.com/documents/nr-reporting-summary-flat.pdf

Ecological, evolutionary & environmental sciences study design

All studies must disclose on these points even when the disclosure is negative.

Study description	This study describes the significance of marine microorganisms utilising hydrogen as an alternate energy source and reveals new mechanisms by which trace gas oxidising marine microorganisms influence the biogeochemistry of global oceans. This study combines culture-dependent and culture-independent based analyses, including genome-resolved metagenomics and thermodynamic modelling to show that diverse marine bacteria consume hydrogen to support growth. Specifically, we demonstrated that microbial communities spanning tropical, temperate and subantarctic waters consume H2 at rates sufficient to support the growth of bacteria with low energy requirements and at environmentally relevant concentrations. Using axenic cultures of the ultramicrobium Sphingopyxis alaskensis, we provide the first demonstration of atmospheric H2 oxidation by a marine bacterium. Concomitantly, using the Tara Oceans dataset, we revealed that the capacity for H2 oxidation as well as carbon monoxide oxidation is

widespread amongst marine bacteria, and of increasing importance in the deeper ocean and in subantarctic waters, with H2 likely supporting mixotrophic growth, and CO supporting long-term bacterial survival.

Metagenomes (n = 14), ex-situ activity (n = 14) and dissolved H2 and CO (n = 14) measurements were prepared using subsamples of homogenised surface water and surface microlayer samples collected from each of the sampling locations (Munida Transect [8], Port Phillip Bay [4] and Heron Island [2]). Growth curves and gas consumption analysis was performed on exponential and stationary phase cultures of Sphingopyxis alaskensis (n = 3), Robiginitalea biformata (n = 3) and Marinovum algicola (n = 3). Quantitative RT-PCR was performed on S. alakensis harvested during exponential (n = 3) and stationary phase (n = 3), performed in technical duplicate.

Research sample	Surface water and surface microlayer samples were obtained from three different sampling locations, eight from the Munida Microbial Observatory Time-Series transect, four from Carrum Beach in Port Phillip Bay, Victoria and two from Heron Island in Queensland. These fourteen samples are representative of diverse marine ecosystems, spanning tropical, temperate, neritic, subtropical and subantarctic waters.<br><br>The existing Tara Oceans global dataset was analysed to provide global comparisons and to further understand the environmental drivers of trace gas oxidation by marine bacteria.It can be obtained from the European Nucleotide Archive. In addition, 1888 bacterial and archaeal MAGs generated by Delmont et al. (2021) were downloaded from https://www.genoscope.cns.fr/tara/. This represents a high quality set of bacterial and archaeal MAGs obtained from the 0.8-2000 µm planktonic cellular size fractions in surface oceans and seas collected by the Tara Oceans expeditions.<br><br>Three distinct marine bacterial species, Sphingopyxis alaskensis, Robiginitalea biformata and Marinovum algicola, were chosen due to the species harbouring hydrogenase classes resembling those identified in the MAGs (namely the group 1l and 2a [NiFe]-hydrogenases). These bacterial species are readily attainable from the authors or via the commercial supplier DSMZ, and are able to be easily grown under laboratory conditions using commercially available growth media.
Sampling strategy	For seawater samples, 1 L water samples were obtained from approximately 0 to 20 cm below the surface using sterile Schott bottles or using a manual glass plate sampler. These samples were then homogenised prior to aliquoting into serum vials in triplicate for ex-situ analysis of dissolved H2 and CO, and microcosm activity experiments. For each sampling location, one set of triplicate samples were autoclaved and used as a control. In addition, a portion of these samples were also used for DNA extraction for metagenomic analysis. For axenic culture experiments, cultures were also grown and analysed in triplicate for each condition. For qRT-PCR experiments, all biological triplicate samples, standards and negative controls were run in technical duplicate. A replication level of three is the standard sample size for environmental microbiological studies, and the results indicate consistency between replicates, with error bars included on all plots to illustrate any variation between replicates No statistical methods were used to predetermine sample size.
Data collection	Samples were collected by different authors as described within the author contribution statement (copied below). All data was recorded digitally and was analysed computationally as follows:<br>- Concentrations of H2 and CO gas for both microcosm and axenic culture experiments were measured using a Valco TGA-6791-W-4U-2 Trace Gas Analyzer<br>- DNA for shotgun metagenomic sequencing was extracted using a DNeasy PowerSoil kit and sample libraries were prepared using a Nextera XT DNA Sample Preparation Kit.<br>- Shotgun metagenomic sequencing was performed on an Illumina NextSeq500 platform at the Australian Centre for Ecogenomics.<br>- Metagenomes were quality controlled using BBDuk and BBMap<br>- Taxonomic profiles were generated from the metagenomes using PhyloFLASH<br>- Metagenome assembled genomes were assembled, binned and analysed using MEGAHIT, MetaBAT2, MaxBin2, CONCOCT, DAS_Tool, RefineM, CheckM, GTDB-Tk, Prodigal and CoverM.<br>- Metabolic annotations were completed using DIAMOND and normalized using the single copy ribosomal proteins available via SingleM.<br>- Phylogenetic analysis was carried out using ClustalW in MEGA11<br>- Environmental driver analysis was carried out in R using the package randomForest<br>- Growth of Sphingopyxis alaskensis was determined using optical density using an Eppendorf BioSpectrophotometer and analysed using GraphPad Prism 9<br>- Quantitative RT-PCR was conducted using a QuantStudio 7 Flex Real-Time PCR system and analysed using GraphPad Prism 9<br><br>Different authors were responsible for field sample collection (G.S., P.A.N., S.L., S.E.M., Y-J.C., A.J.K., P.L.M.C.), gas chromatography measurements (Z.F.I., G.S., T.J.), biogeochemical analysis (G.S., P.M.L., P.A.N., C.G.), thermodynamic modelling (P.M.L, C.G., F.B., P.M.L.C), culture based work (Z.F.I., T.J., G.S., T.J.W., R.C., C.G.), metagenome analysis (R.L., G.S., S.L., C.G.) and environmental driver and phylogenetic analysis (R.L., G.N., C.G.).
Timing and spatial scale	Field sample collection started on 20/03/2019 and finished on 23/07/2019, with Port Phillip Bay samples obtained on 20/03/2019, Heron Island samples obtained on 09/07/2019 and the Munida Transect samples obtained on 23/07/2019. Field samples were collected on the same day for each sampling location and were collected <1 m below the surface of the water. Ex situ microcosm activity assays were carried out immediately post field sample collection for each field site.<br><br>Growth curve and gas chromatography measurements for Sphingopyxis alaskensis, Robiginitalea biformata and Marinovum algicola were collected between 18/11/2019 and 23/12/2020; note extended timescale was due to the impact of COVID-19 related lockdowns in Melbourne, Australia. Quantitative RT-PCR analysis on S. alaskensis was carried out between 28/09/2020 and 29/10/2020.
Data exclusions	The metagenomes were filtered to remove reads corresponding to major organisms detected in the extraction control. One timepoint in the ex-situ gas chromatography measurements was removed due to machine error.
Reproducibility	All experiments and measurements undertaken were successful, and replicates were consistent within so we did not attempt to repeat the experiments beyond the technical and biological replications used within each experiment.

Randomization

Samples that were obtained in the field were returned to the laboratory, homogenized and sub-sampled for the various treatments for the ex situ microcosm and gas chromatography experiments. For axenic culture experiments, bacterial cultures were independently inoculated into triplicate flasks for growth, gas chromatography and qRT-PCR experiments, leading to three biologically distinct populations of bacterial cells per treatment condition.

Blinding

Blinding was not relevant to our study as it relates to solely microbial processes and experimental data was collected electronically and analysed digitally.

Did the study involve field work? ☒ Yes ☐ No

Field work, collection and transport

Field conditions

For the Port Phillip Bay and Heron Island samples, these were collected manually from shallow water (~1m deep) on fine weather days. Carrum Beach is situated in Port Phillip Bay, ~ 50 km from the central business district of Melbourne, Victoria, Australia. The site is accessible to general public. Port Phillip Bay is a relatively large, shallow embayment (1930km2, mostly < 8 m deep, max depth 24 m) which provides protection to its beaches from severe weather. Seawater temperature ranged from 13.6 to 20.3°C based on historical record (https://www.seatemperature.org/australia-pacific/australia/port-phillip.htm). Heron Island is a natural coral cay located approximately 80km north east of Gladstone, Queensland, Australia. It is accessible to the public by boat or helicopter. Seawater temperatures range from 21.5 to 27.3°C based on historical record (https://seatemperature.info/heron-island-water-temperature.html)/. The Munida Microbial Observatory Time Series is a surface water transect that extends 65 km off the coast of Otago, New Zealand, and includes neritic, subtropical and sub-Antarctic surface waters (https://www.otago.ac.nz/mots/about/). It is only accessible by a research vessel moored at Otago University, New Zealand. Surface water temperatures range from 9 to 13°C based on historical records (https://www.st.nmfs.noaa.gov/copepod/time-series/nz-10101/).

Location

Eight samples were collected from across the Munida Microbial Observatory Time-Series transect (Otago, New Zealand) on 23/07/2019, in calm weather, on the RV Polaris II. Eight equidistant stations were sampled travelling east, ranging from approximately 15 km to 70 km from Taiaroa Head (~45.7828° S, 170.7333° E to ~45.8°S, 171.6° E). At each station, water was collected at 1 m depth using Niskin bottles. Four samples were also collected from the temperate Port Phillip Bay at Carrum Beach (38.0765° S, 145.1206° E, Victoria, Australia) on 20/03/2019 and two were collected from the tropical Heron Island (23.4423° S, 151.9148° E, Queensland, Australia) on 9/7/2019. At both sites, near-shore surface microlayer and surface water samples were collected in the subtidal zone (water depth ca. 1 m; sampling depth of ~20 cm). At Port Phillip Bay, two samples were also collected at 7.5 km and 15 km east of the mouth of the Patterson River, labelled 'Intermediate' and 'Centre' respectively. All samples were collected at sea level.

Access & import/export

Suitable footwear, gloves and equipment, including pre-sterilised Schott bottles, were used during the sampling process to minimize anthropogenic effects for all samples collected. Water samples were collected in compliance with Australian biosecurity regulations, with those from the Munida Transect also subject to New Zealand biosecurity laws.

Disturbance

The study was minimally disruptive to the sampling locations as water was only collected from the surface and surface microlayer using Schott bottles and manual glass plate samplers, respectively. In total, <20 L of water was collected from the Munida Transect, <15 L of water was collected from Port Phillip Bay and <10 L of water was collected from Heron Island. Disturbance was negligible as small volumes of samples were collected from each sampling location in a non-invasive manner.

Reporting for specific materials, systems and methods

We require information from authors about some types of materials, experimental systems and methods used in many studies. Here, indicate whether each material, system or method listed is relevant to your study. If you are not sure if a list item applies to your research, read the appropriate section before selecting a response.

Materials & experimental systems

n/a	Involved in the study
☒	☐ Antibodies
☒	☐ Eukaryotic cell lines
☒	☐ Palaeontology and archaeology
☒	☐ Animals and other organisms
☒	☐ Clinical data
☒	☐ Dual use research of concern

Methods

n/a	Involved in the study
☒	☐ ChIP-seq
☒	☐ Flow cytometry
☒	☐ MRI-based neuroimaging
